# X or Y Cancer: An Extensive Analysis of Sex Differences in Lung Adenocarcinoma

**DOI:** 10.3390/curroncol30020107

**Published:** 2023-01-18

**Authors:** Raneem Yaseen Hammouz, Magdalena Orzechowska, Dorota Anusewicz, Andrzej K. Bednarek

**Affiliations:** Department of Molecular Carcinogenesis, Medical University of Lodz, 90-752 Lodz, Poland

**Keywords:** adenocarcinoma, sex, metabolism, hormones, menopause, immune, biomarkers

## Abstract

**Background:** Cellular metabolism is a tightly controlled process during which cell growth and survival are maintained. Lung cancer is a disease with clear sex differences, where female patients have better survival rates than males. Evidence of sex differences is demonstrated in cancer risk, prognosis and response to different therapies, yet a sex-specific approach to cancer studies is not widely considered. These different tumour characteristics attributed to sex that impact disease outcome, including constitutional genetic and somatic molecular differences, make it essential to assess viral and hormonal influences. **Methods:** In silico analysis of lung adenocarcinoma (LUAD) TCGA data, including K-means clustering algorithm, dimensional reduction with principal component analysis and differential expression analysis using EdgeR (*p* < 0.05), were used to explore some robust sex differences in LUAD that exist in core signalling pathways and metabolic processes between males and females. The correlation of differentially expressed genes (DEGs) expression with immune abundance in the LUAD cohort was analysed on TIMER2.0 and adjusted by tumour purity utilising Cox proportional hazard. Multiple factorial analysis heatmap visualisation was used to examine endogenous steroid hormonal effects on LUAD patients with different smoking status and age groups. **Results:** We found 161 DEGs showing key differences in regulation of immune system and cellular homeostasis, key elements of divergent cancer progression, between the two sexes. We also found male and female LUAD patients to favour different metabolic intermediates for energy production to support tumourigenesis. Additionally, high levels of Tregs accompanied by DEGs correlated with better LUAD prognosis, and circulating hormonal transcriptional targets affect proliferation and progression in males and females differently. Finally, we examined the role of oestrogen protection in men and pre-/postmenopausal women. **Conclusions:** Further studies should focus on sex-specific changes and investigate sex-specific gene regulatory networks of these DEGs. Several lifestyle factors, including tobacco smoking and diet, differ between males and females. These factors might affect metabolic pathways and can influence the activity of epigenetic regulators, resulting in significant global epigenetic changes.

## 1. Introduction

Lung cancer is the major cause of cancer-related deaths worldwide, affecting more males than females, with the latter having better survival [1]. Additionally, data indicate more females than males associated with non-smoking lung cancer. Sex hormones and biological sex influence lung structure, development and physiology. Even though sex differences are evident in tumour incidence and mortality across age amongst different cancer types, sex is usually an unexplored variable in cancer studies or controversially discussed. Sex disparities are also observed in cancer mortality. Following age adjustment, the mortality rate of all cancer sites combined is 214 for males and 149 for females per 100,000 [2]. These differences involve biological, physiological, hormonal as well as genetic factors. Therefore, since genomic features associate with cancer aetiology, prognosis and treatment response, they could result in differential effects in both sexes—male and female. Smoking is undoubtedly connected with incidence and mortality of lung cancer. Females seem to be biologically more susceptible to the effects of carcinogens than males, implying the possibility that females might metabolise smoke carcinogens differently [3]. Furthermore, there are significant sex differences in therapeutic response, toxicity and efficacy for several cancer types. Exploring molecular mechanisms that could possibly partake in the different clinical manifestation in both sexes is vital as it may influence cancer in different ways.

Moreover, lung adenocarcinoma (LUAD) is considered a different disease in males and females, strongly influenced by oestrogen [4]. Oestrogen metabolites cause DNA oxidative damage since they generate reactive oxygen species (ROS), and, by inducing oestrogen-expression-dependent genes, the oestrogen receptor (E2/ER) complex promotes non-small-cell lung cancer (NSCLC) cell cycle progression and proliferation. Steroid hormone receptors, such as oestrogen receptor alpha (ERα) and oestrogen receptor beta (ERβ) and progesterone, have been detected in normal and tumour lung tissue, linked with survival [4]). In NSCLC, smoking was associated with higher cytoplasmic ERα and ERβ but lower progesterone expression. Females had lower ERβ nuclear expression compared to men. The difference in ERβ nuclear expression provides insight for prevalence of lung cancer in females. ERα and ERβ mediate cellular responses to oestrogen through nuclear and cytoplasmic compartments [5]. ERα and ERβ signal in opposite ways depending on ligand and response elements. ERα promotes gene transcription through binding with oestrogen-responsive elements and activator protein (AP-1) enhancer elements in the promoter of target genes in the cytoplasm, while ERβ inhibits transcription of AP-1 sites located in the cell nucleus. Females with higher ERβ nuclear expression were less susceptible to hormone-related lung cancer as its expression was lower in postmenopausal women compared to premenopausal women, possibly due to the lower levels of oestrogen [6]. No statistical significance was found between ERβ nuclear expression and sex; however, smoking was associated with increased cytoplasmic ERα and ERβ, suggesting a link to ER phosphorylation through smoking. This runs parallel with the fact that lung cancer smokers who might have higher expression of cytoplasmic ERα and ERβ have worse survival compared to non-smokers. Therefore, lower levels of ERβ nuclear expression in women might support the oestrogen protection hypothesis, while increasing cytoplasmic ERα and ERβ might be due to the effect caused by smoking disrupting hormone pathways [4]. Additionally, oestrogen can directly stimulate transcription of oestrogen-related genes in the nucleus of lung cells and ER, mainly ERβ, which are present and functional in lung and tumour cells. Oestrogen can also transactivate growth factor signalling pathways, including epidermal growth factor (EGF) pathway [5].

There is a difference in the mechanism of oestrogen receptor action in premenopausal, postmenopausal women and men. Men have lower circulating oestradiol (E2) concentrations compared to premenopausal women. The ovaries of healthy premenopausal women produce circulating 17β-oestradiol, which acts on distant target tissues through conversion of androstenedione to estrone. In postmenopausal women and men, E2 is produced in extragonadal sites, such as adipose tissue and others, from aromatisation of circulating testosterone, making testosterone a circulating pro-hormone locally converted to E2 acting on ER or converted into a second oestrogen (5alpha-androstane-3beta,17beta-diol), which acts on ER. In females, androgen receptor (AR) activation is weaker; thus, AR is less important unless concentrations increase to those levels of males; then, it might also lead to metabolic disturbances. Evidence of metabolic differences in relation to oestrogen protection indicates that postmenopausal women have increased risk of developing type 2 diabetes due to higher plasma levels of E2 associated with higher levels of testosterone [7]. AR induces telomerase expression in primary hematopoietic stem cells (HSCs) through aromatase-dependent conversion of testosterone into oestrogen and ERα activation, whereas oestrogens are pro-angiogenic and enhance endothelial cell proliferation and migration mediated by diffusible factors, such as vascular endothelial growth factor A (VEGFA) and platelet activating factor, both secreted regularly in response to oestrogen by cancer cells. Only in male endothelial cells, where there are higher levels of AR expression, androgens stimulate endothelial cell migration and angiogenesis through VEGFA and, through VEGFR2, promote proliferation [8].

Here, we provide molecular insights into the sex-specific genetic and genome-wide impact on LUAD, illustrating how males and females seem to favour different metabolic intermediates for energy production to support tumorigenesis, influencing LUAD biology. Our results show dissimilarities in immune response and cellular homeostasis between the sexes, as well as different signalling pathways and metabolic processes between LUAD male and female patients. We also show different hormonal transcription target regulation among different LUAD smoking groups and present different regulation of sex-specific differentially expressed genes (DEGs) according to different age groups in females compared to males, including the effect of oestrogen protection in premenopausal women.

## 2. Materials and Methods

### 2.1. Data Collection

**Figure 1:**
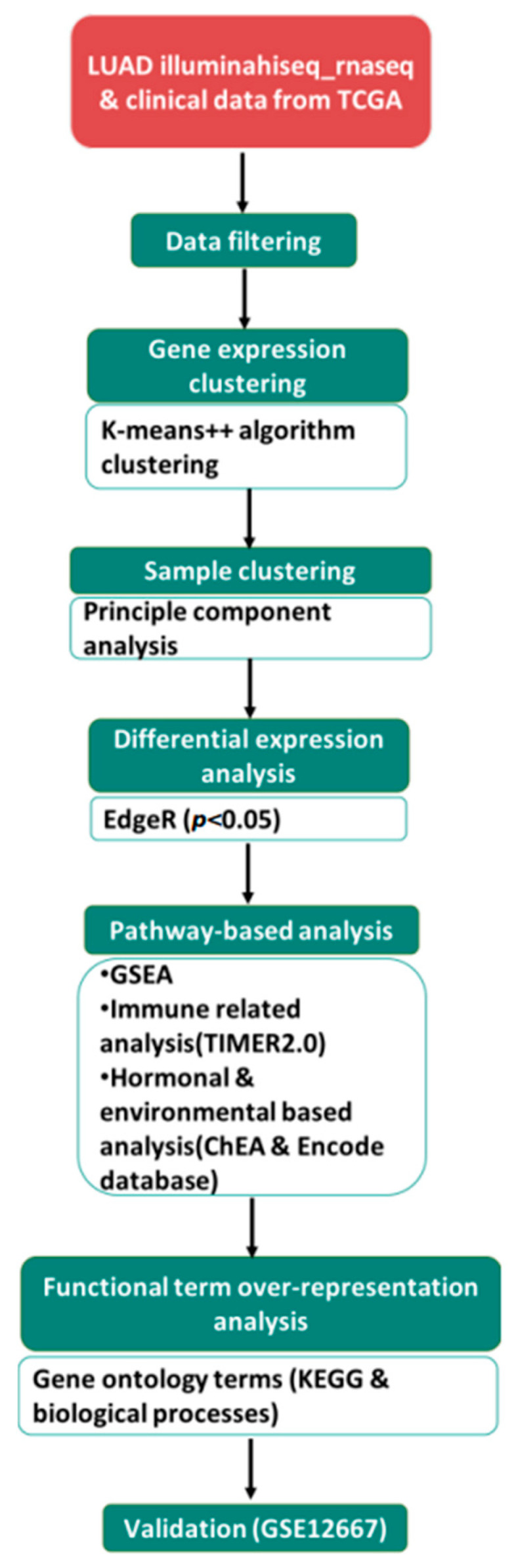
Flowchart of the data analysis process. illuminahiseq_rnaseq and patient clinical data were downloaded from TCGA. Following data filtering, partitioning modified K-means clustering algorithm was performed using ExpressCluster. Principal component analysis (PCA) using R was used to explore the 6 patient subgroups (FN, FC, FR, MN, MC and MR), assessing the variation across each category according to sex. EdgeR was used to explore differential expression, yielding 161 DEGs (*p* < 0.05). Pathway analysis was also performed (refer to the methodology section for the detailed approach). Validation of DEGs was performed on GSE12667 probes.

Collection of the expression profile of LUAD TCGA data has been previously described [9]. For validation, gene chip GSE12667 of LUAD with its clinical manifestation data was also downloaded from Genome Expression Omnibus (GEO) database [10]. The study included 497 patients from TCGA and 75 probes from GEO databases. There were 229 males and 268 females; further grouping according to smoking status included a permissible value of 1 for lifelong non-smokers (NS) and 2 for current smokers (CS) and 3 and 4 for reformed smokers (RS). Additionally, phenotypes regarding menopause were classified as perimenopausal: females under 55 years of age, postmenopausal: females over 55 years of age and males under 70 years of age were included. A series of clinical traits for TCGA patients are shown in Supplementary Table S1; additionally, an overview of the analysis workflow is shown in Figure 1.

### 2.2. Defining a Gene List of Interest and Identifying Regulatory Networks

To find common and unique expression gene profiles showing differentiation between the patient subgroups, we used ExpressCluster software [11]. Clustering was performed by applying K-means++ algorithm, Z-norm signal transformation and rank correlation distance metric. Profiles indicating contrast between female: FN, FC; and male: MN, MC subgroups were considered significant. Principal component analysis (PCA) was used to explore the 6 patient subgroups, including reformed smokers, assessing the variation across each category according to sex. EdgeR [12] was used to confirm the differentially expressed genes (DEGs) between sexes. Benjamini–Hochberg (BH) method on the *p*-values < 0.05 was used to control the false discovery rate (FDR), and generalized linear model glmFit to minimise error function was used to determine differential expression.

Gene set enrichment analysis (GSEA) [13] was also employed to determine whether the identified DEGs showed significant functions between the patient subgroups using all Human MSigDB gene set collection and to identify the enriched genes between the 3 phenotypes (perimenopausal and postmenopausal females, and males). Datasets and phenotype label files were created (defined either as females above 55, females under 55 or males under 70) and loaded onto the GSEA software (v3.0). ChIP-X enrichment analysis (ChEA) database [14] and the Encode database [15] were used to search the transcription factor targets that could regulate the oestrogen (ESR1 and ESR2) and androgen receptors (AR). MitoCarta database [16] was used to examine specifically the mitochondrial proteome to filter a list of mitochondrial proteins detected in our DEGs.

### 2.3. Gene Enrichment Analysis of DEGs

Enrichment analysis was performed as previously described [9] according to the expression data for males and females to all 20,502 genes in terms of all the Molecular Signatures Database (MSigDB) collections [17]. Functional classification was conducted after literature and database search as well as gene ontology (GO) enrichment analysis following GSEA and using ShinyGO [18]; false discovery rate FDR < 0.05 was used for DEGs in both phenotypes separately.

### 2.4. Correlation of DEGs with Immune Infiltrates

The correlation of our DEGs expression with immune abundance in our cohort was analysed using Tumor Immune Estimation Resource (TIMER) [19,20,21] and adjusted by the tumour purity. First, we used the estimation module on our cohort employing all 497 samples to infer the abundance of tumour-infiltrating immune cells (TICC). We then accessed the Outcome module to infer the correlation with immune gene signature from CIBERSORT, CIBERSORT-ABS and TIMER, of which 22 DEGs were used for further analysis using the LM22 gene signature. The immune infiltration levels of the 22 DEGs were used to explore the clinical relevance with the TIIC subsets along with clinical covariates (sex). Purity-adjusted partial Spearman’s rho values and associated *p*-values were generated, representing the degree of correlation. We utilised Quantiseq to evaluate the impact of immune factors and the expression of 22 immune-related DEGs on LUAD prognosis. Quantiseq is a validated deconvolution-based algorithm that estimates the absolute proportions of relevant immune cell types from RNA-seq [22].

### 2.5. Statistical Analysis

To confirm the DEG obtained, EdgeR was used as stated in Section 2.2. Regarding multifactorial analysis (MFA), heatmap visualization for median expression values was used and gplots [23], R package, row clustering according to Pearson’s distance metric and complete agglomeration method were used.

### 2.6. Association Analysis between DEGs and Patient Prognosis

TIMER2.0 gene outcome module was employed to evaluate the gene expression association on patient survival in relation to sex utilising Cox proportional hazard model for association evaluation, validating the results from GEPIA2 analyses [24].

## 3. Results

### 3.1. Genetic Differences Analyses

#### 3.1.1. Sex-Specific Molecular Signature of LUAD

**Figure 2:**
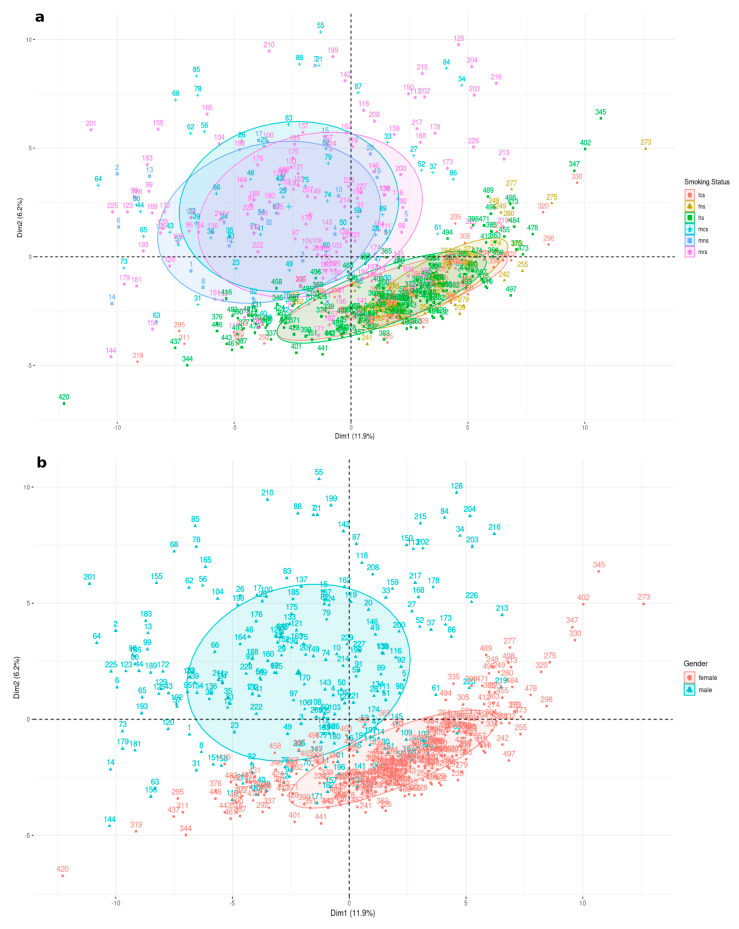
Principal component analysis performed on expression data for 161 genes for all 6 LUAD subgroups. Subgroups clusters are represented on the factorial plane by coloured ellipses reflecting their association with 11.9% and 6.2% of the total variability for the first two dimensions. (**a**) represents all six groups: male never smokers (MNS), current smokers (MCS), female never smokers (FNS) and female current smokers (FCS), while (**b**) represents males and females collectively.

K-means clustering, on all 20,501 genes downloaded from TCGA according to their sexual phenotype, enabled identification of 161 genes, contributing to a clear distinction of LUAD patients according to sex, further confirmed using EdgeR with an adjusted *p*-value < 0.05. We found 71 upregulated genes and 90 downregulated genes (*p* < 0.05). The magnitude of the differential expression changes was visualized with a fitted model MD plot (Supplementary Figure S1 and Table S2). As shown in Figure 2, these 161 specific DEGs are divided into female never smokers, current smokers and reformed smokers (FNS, FCS and FRS) and male never smokers, current smokers and reformed smokers (MNS, MCS and MRS) clusters according to their molecular signature differences. Further GO analysis via GSEA and ShinyGO indicates DEGs to be involved in proliferation, apoptosis, epithelial mesenchymal transition (EMT), immune and metabolic responses as well as transcription factor targets for Wnt, Notch, TGF-β and ErbB signalling pathways.

**Figure 3:**
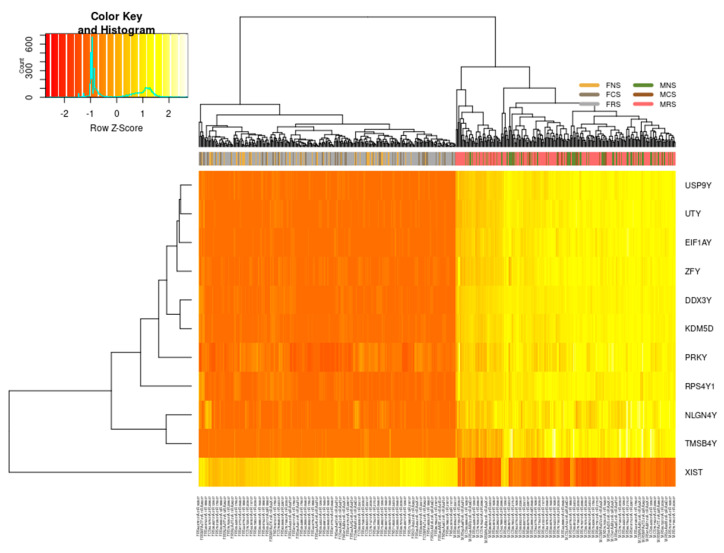
Differentially expressed genes encoded on the sex chromosomes in LUAD cohort. On the right, all 10 genes: *USP9Y*, *UTY*, *EIF1AY*, *ZFY*, *PRKY*, *NLGN4Y*, *TMSB4Y*, *DDX3Y*, *KDM5D*, *RPS4Y1* are upregulated in males (*n* = 229) regardless of smoking status (MNS, MCS, MRS) or age (range 38–88 years of age), and on the left are downregulated in females (*n* = 268), likewise irrespective of smoking status (FNS, FCS, FRS) or age (39–87 years of age).

Regardless of smoking status, stage or age, we identified 11 DEGs in our cohort that differ in their expression pattern between females and males (Figure 3). These differences are the result of sexual differentiation involving genetic and epigenetic mechanisms. *USP9Y*, *UTY*, *EIF1AY*, *ZFY*, *PRKY*, *NLGN4Y*, *TMSB4Y*, *DDX3Y*, *KDM5D* and *RPS4Y1* were all upregulated in males (MNS, MCS, MRS) with significant fold changes log_2_FC > 6 and only *XIST* downregulated in females (FNS, FCS, FRS) with significant fold changes log_2_FC < −5. We confirmed that these 11 genes differentiate both sexes in relation to LUAD carcinogenesis also by verifying whether other X- or Y-linked genes, termed “control genes”, demonstrate a similar pattern (Supplementary Figure S2). We did not find a difference in expression of these “control” genes between the two phenotypes, thus confirming specificity in regard to LUAD rather than a general sex-specific difference.

Supplementary Table S3 lists the related gene ontology (GO) terms for nine of the upregulated DEGs in males. Six upregulated DEGs in males are involved in cell cycle regulation: helicase *DDX3Y*, and in cell differentiation; *EIF1AY* is involved in RNA transport and translational factors; *KDM5D* chromatin and transcription regulation and encodes lysine demethylases (epigenetic modifier) as well as in regulation of androgen receptor signalling pathway; *UTY* in dioxygenase activity and histone demethylase activity (H3-K27 specific); *ZFY* transcriptional activation and *RPS4Y1* in RNA binding and ribosomal assembly. *USP9Y* is an essential component of the BMP/TFG-β signalling cascade, *NLGN4Y* in cell–cell interactions and cell adhesion and *TMSB4Y* in cytoskeletal organisation. Finally, *PRKY*, a pseudogene, is involved in protein phosphorylation and ATP binding, while long non-coding RNA (lncRNA) *XIST* is a major effector of the X-inactivation process via histone deacetylation and enriching repressive chromatin marks.

#### 3.1.2. Altered Warburg Metabolism Seems to Inhibit AMPK but Activate mTOR and MAPK Signalling in Our Female Cohort

Our results seem to indicate the “Warburg effect” is mostly active in females, especially with elevated expression of hexokinase and phosphofructokinase (*PFKL*, *HK1*) compared to males. We also found mTOR, MAPK, PI3K-Akt and RAS signalling pathway genes to have higher expression mostly in females and AMPK signalling pathway genes with elevated expression mostly in males (Table 1). Additionally, some FOXO signalling genes in our cohort are involved in cell cycle control or cell signalling (*CDKN1A*, *CDKN1B*, *INSR*, *IRS2*, *EIF4E*), metabolism (*SREBF1*, *SIRT1*, *PPARGC1A*, *APOE*), DNA repair (*GADD45A*) and immune responses (*ARG1*, *ULK2*). Since FOXO signalling genes were upregulated with higher expression mostly in our male cohort while downregulated with lower expression in our female cohort, we speculate that, in our male cohort, FOXO signalling seems to maintain cellular energy homeostasis. Furthermore, we found glycolysis, glucogenesis and fatty acid/lipid synthesis enriched in females, where downregulated genes *ACSS1*, *ACSS2*, *IL4I1* and *XIST* were expressed at a higher level compared to males. Meanwhile, oxidative phosphorylation genes *ATP1B1*, *ATP12A*, *ATP6V0A4*, *ATP6V1E1*, *ATP6V1G1*, *NDUFV2* and pyruvate metabolism *PDHB* genes demonstrated higher expression in males.

#### 3.1.3. Metabolism of Xenobiotic by Cytochrome p450 and DNA Repair Seem to Be Upregulated in Our Male Cohort

We found several xenobiotic and drugs processing genes, including ATP-dependent transporter of the ATP-binding cassette (ABC) (Supplementary Figure S3), to be upregulated with higher expression in our male cohort: *MGST1*, *AKR1C3*, *ALDH3B1*, *TAT*, *AKR1C1*, *AKR1C2*, *ITIH4*, *IGFBP1*, *GSR*, *ABCC2*, *PGD*, *ENPEP*, *MCCC2*, *GABARAPL1*, *GSTM4*, *GSTO2*, *GSTA2*, *PTGES3*, *AHCY*, *CYP2C8* and *CBR1* while a few downregulated with higher expression in females: *APOE*, *PINK1*, *ALDH1A3*, *GSTA3*, *PPARD* and *TGFB2*, some of which seem to participate in conversion of benzoapyrene, nicotine, naphthalene and aflatoxin. Aryl hydrocarbon receptor (AHR) signalling pathway seemed to be altered in both sexes (Table 1).

Moreover, genes involved in ROS pathway were upregulated and had higher expression level in our male cohort, including *ABCC1*, *G6PD*, *GLRX2*, *GSR*, *HBXIP*, *MGST1*, *NDUFB4*, *PRDX4*, *PRDX6*, *SELS* and *TXNRD2*.

**Figure 4:**
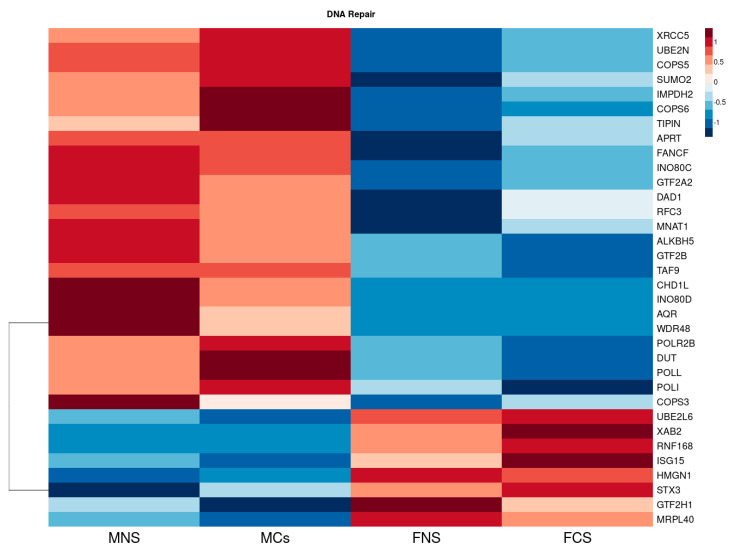
Heatmap reflecting the differential median expression across all the samples using the DNA repair DEGs in LUAD cohort between male never smokers (MNS), current smokers (MCS), female never smokers (FNS) and female current smokers (FCS).

Additionally, we found several DNA repair genes to be upregulated with higher expression in our male cohort (Figure 4), including *XRCC5*, *UBE2N*, *COPS5*, *SUMO2*, *IMPDH2*, *COPS6*, *TIPIN*, *APRT*, *FANCF*, *INO80C*, *GTF2A2*, *DAD1*, *RFC3*, *MNAT1*, *ALKBH5*, *GTF2B*, *TAF9*, *CHD1L*, *INO80D*, *AQR*, *WDR48*, *POLR2B*, *DUT*, *POLL*, *POLI* and *COPS3*. However, in our female cohort, we only observed a few downregulated DNA repair genes to have higher expression than in males, including *UBE2L6*, *XAB2*, *RNF168*, *ISG15*, *HMGN1*, *STX3*, *GTF2H1* and *MRPL40*.

#### 3.1.4. Expression of 22 DEGs Strongly Associates with Tumour Infiltration Abundance of Immune Cells

We attempted to explore the relationship between the mRNA expression of our DEGs and immune cell infiltration levels across all 497 samples using TIMER2.0 (as described in Section 2.4) (Supplementary Figure S4). We observed that these genes present significant association with infiltrating levels of lymphocytes, macrophages, neutrophils, DCs and T-regulatory cells (Table 2). Our results suggest the potential role of these 22 DEGS in regulating immune response in LUAD.

These genes mostly correlated positively with T-regulatory cells (COR, −0.122 to 0.516; *p* < 0.0001), B lymphocytes (COR, −0.063 to 0.74; *p* < 0.0001), CD8+ T cells (COR, −0.15 to 0.63; *p* < 0.05) and macrophages (COR, −0.19 to 0.445; *p* < 0.05) and mostly correlated negatively with CD4+ T cells (COR, −0.236 to 0.292; *p* < 0.05) and showed the least correlation with neutrophils (COR, −0.142 to 0.234; *p* < 0.05) and DC (COR, −0.204 to 0.141; *p* < 0.05).

This indicates that these genes were positively and a few negatively related to tumour-associated lymphocytes, macrophages, DC and neutrophils in the LUAD microenvironment.

#### 3.1.5. High Level of Infiltrated Regulatory T Cells Accompanied by DEGs Correlates with Better LUAD Prognosis

**Figure 5:**
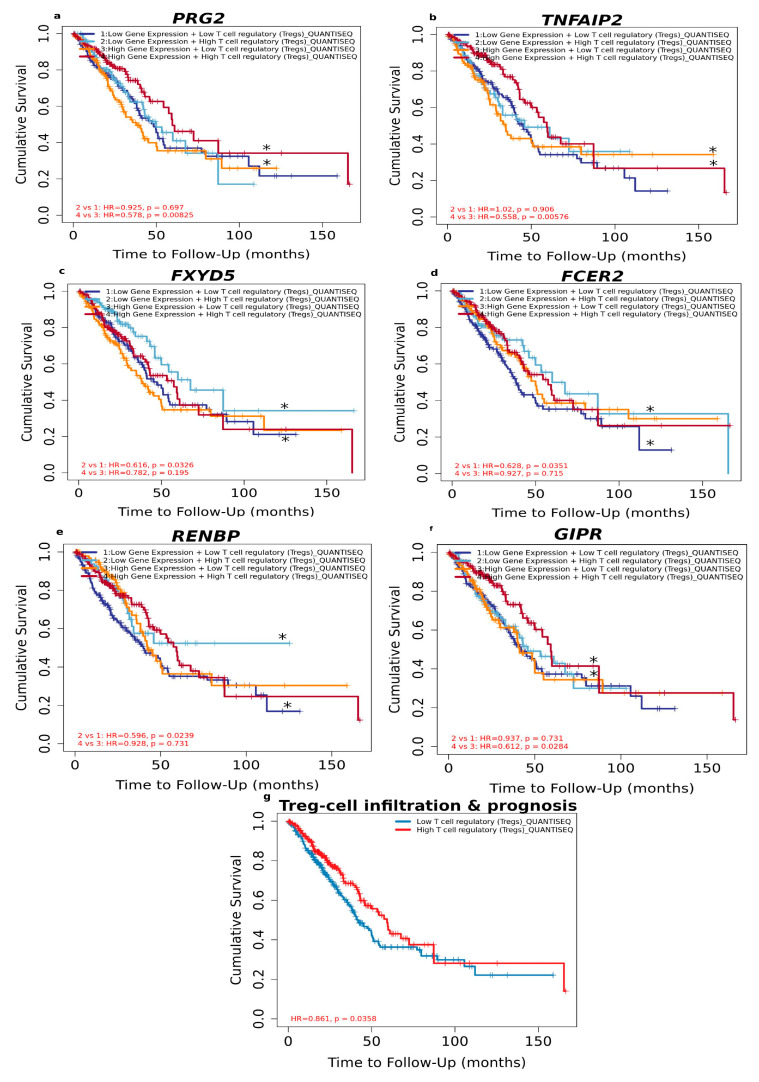
Overall survival analysis for combining the expression of single DEGs and Tregs in LUAD patients. (**a**) *PRG2*, (**b**) *TNFAIP2*, (**c**) *FXYD5*, (**d**) *FCER2*, (**e**) *RENBP*, (**f**) *GIPR*, (**g**) relationship between Treg-cell infiltration and prognosis of LUAD according to sex based on Quantiseq algorithm. (*) indicates *p*-value < 0.05 was considered statistically significant.

By analysing different regulatory T cells’ (Tregs) cell infiltration levels and LUAD prognosis, we found that patients with high Tregs infiltration levels had better prognosis, as verified by Quantiseq (Figure 5). We, therefore, choose to focus on Treg infiltration levels since Tregs are vital for preventing autoimmunity and regulating inflammation. Therefore, we evaluated the prognostic efficacy of the combination of Tregs and expression patterns for single DEGs in regard to sex (Figure 5, Supplementary Figure S5). Analysis of the tumour immune microenvironment could provide accurate personalized treatment plans for patients.

As shown in Figure 5, there was no significant relationship between Tregs and prognosis regarding the high expression level of *FCER2, FXYD5* and *RENBP*. Neither was there a significant relationship between Tregs and prognosis regarding the low expression level of *GIPR, TNFAIP2* and *PRG2*. However, under low expression of *FCER2* (HR = 0.628, *p* = 0.0351)*, FXYD5* (HR = 0.616, *p* = 0.0326) and *RENBP* (HR=0.596, *p* = 0.0239) and high Tregs level predicted favourable prognosis, whereas high Treg level predicted favourable prognosis under high expression of *GIPR* (HR=0.612, *p* = 0.0284), *PRG2* (HR = 0.578, *p* = 0.00825) and *TNFAIP2* (HR = 0.558, *p* = 0.00576).

These results suggest that the above-mentioned DEGs are independent favourable prognostic biomarkers and combining them with Tregs would allow a more effective role in prognosis prediction and personalised therapy for LUAD.

### 3.2. Hormone-Related Analyses

#### 3.2.1. Circulating Hormonal Transcriptional Targets Affect LUAD Tumour Proliferation and Progression

**Figure 6:**
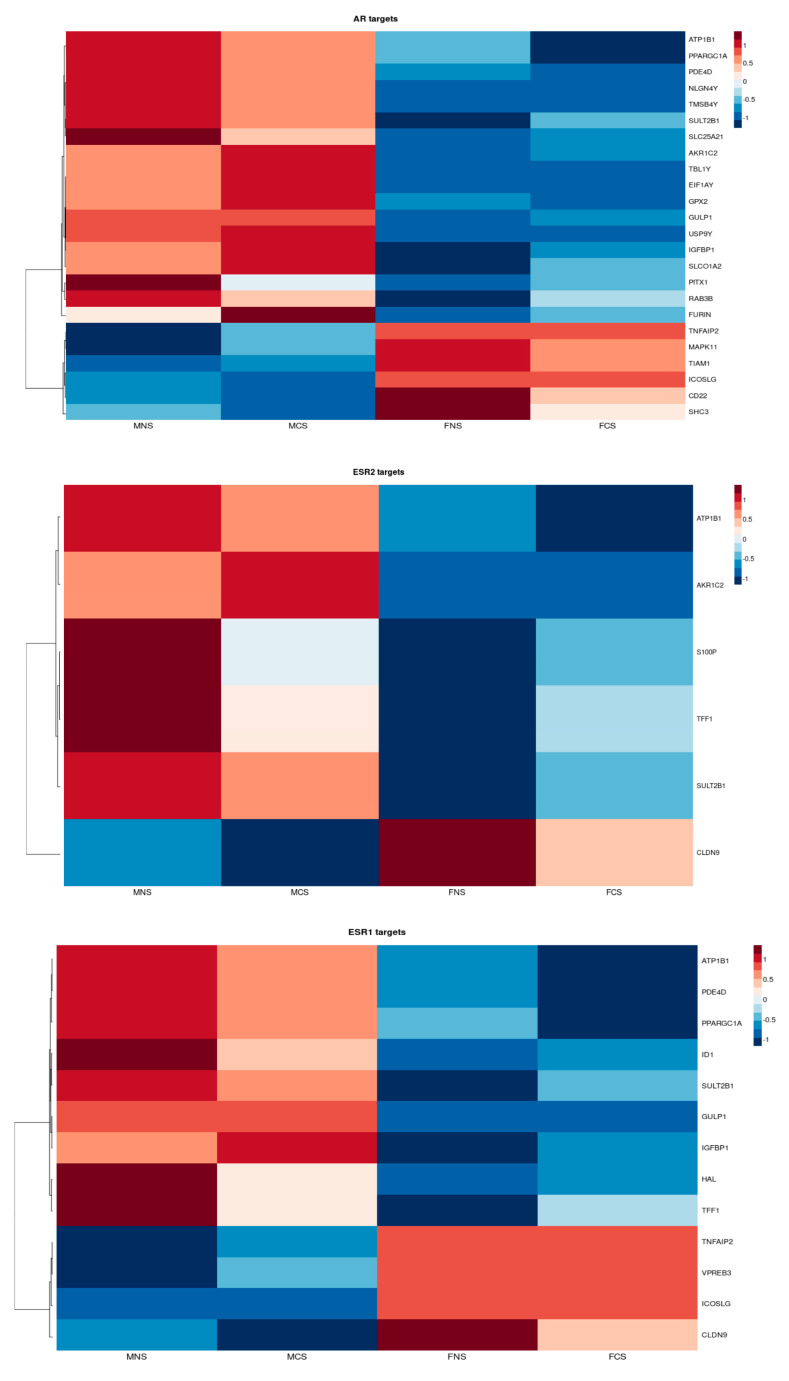
Heatmap reflecting the differential median expression across all the samples according to DEGs encoding androgen and oestrogen transcriptional target in LUAD cohort between male never smokers (MNS), current smokers (MCS), female never smokers (FNS) and female current smokers (FCS).

To examine the steroid hormone receptors in LUAD, we investigated the role of DEGs encoding oestrogen receptor-α (ESR1) and β (ESR2) and androgen (AR) transcription targets in both phenotypes. Our results clearly show sex bias (Figure 6). *ATP1B1, CLDN9, SULT2B1* and *TFF1* are both ER-α and ER-β transcription targets; *ARK1C2* and *ATP1B1* are both AR and ER-β transcription targets. Finally, *GULP1, ICOSLG, IGFBP1, PDE4D, PPARGC1A, SULT2B1* and *TNFAIP2* are AR and ER-α transcription targets.

We found expression of transcription targets *ATP1B1*, *ARK1C2*, *GULP1*, *HAL*, *ID1*, *IGFBP1*, *PDE4D*, *PPARGC1A*, *TFF1*, *SULT2B1*, *NLGN4Y*, *TMSB4Y*, *SLC25A21*, *TBL1Y*, *EIF1AY*, *GPX2*, *GULP1*, *USP9Y*, *IGFBP1*, *SLCO1A2*, *PITX1*, *RAB3B* and *FURIN* to be upregulated in males (MNS, MCS) and downregulated in females (FNS, FCS) in contrast to expression of *TNFAIP2*, *VPREB3*, *ICOSLG*, *CLDN9*, *MAPK11*, *TIAM1*, *CD22* and *SHC3*.

These differences in gene expression amongst the groups seem to be partly attributable to the differential presence of the levels of endogenous sex-related hormones rather than smoking status.

These hormonal transcriptional targets associated mainly with proliferation (*ARK1C2*, *FURIN*, *ID1*, *MAPK11*, *PPARGC1A*, *TFF1*, *ICOSLG*), PID HIF1 TF pathway (*FURIN*, *IGFBP1*), EMT (*ID1*, *TIAM1*, *HAL*), apoptosis (*PPARGC1A*, *ATP1B1*, *SLCO1A2*), AMPK-signalling pathway (*PPARGC1A*), adhesion (*SHC3*, *ATP1B1*, *CLDN9*, *ICOSLG*, *NLGN4Y*, *TBL1Y*) and ROS (*GPX2*).

#### 3.2.2. Association of Oestrogen Protection in Males and Premenopausal and Postmenopausal Females

**Figure 7:**
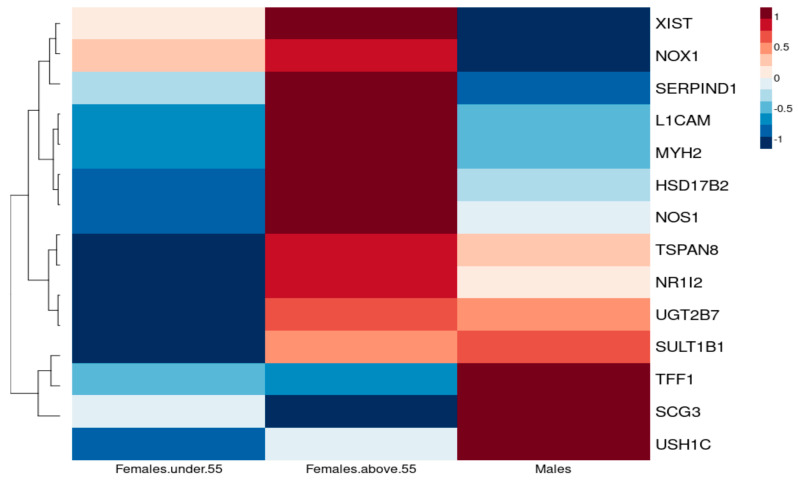
Heatmap reflecting the differential median expression across all the samples, illustrating the individual genes that are differentially expressed between premenopausal (under 55 years of age), postmenopausal females (over 55 years of age) and males.

NSCLC cancers exhibit higher incidence and a more aggressive pattern in males and postmenopausal females compared to premenopausal females [8]. Furthermore, lung cancer postmenopausal women seem to show significant enhanced survival in comparison to premenopausal women, and some report even survival advantages over men [4]. To explore the potential protective effect of oestrogen, we compared expression of common DEGs with a log_2_FC > 1.5 in three groups: 43 females below 55 years of age, 215 females above 55 years of age and 138 males under the age of 70 (Figure 7).

We suspect that pathways responsible for cancer development for *TSPAN8, UGT2B7, SULT1B1, L1CAM* and *MYH2* differ according to hormonal status as they vary in females under 55 years of age (pre- and perimenopausal women) and postmenopausal women, whereas, for postmenopausal women, regulation of *TSPAN8*, *UGT2B7* and *SULT1B1* could possibly imply that non-sex-hormone mechanisms are involved since their expression is similar to that in males. *TFF1*, however, seems to show a difference in sex rather than menopausal status.

Indeed, increased expression of *TSPAN8* has been associated with increased proliferation, migration and angiogenesis induction [25], further supporting the protective role of oestrogen in premenopausal women, whereas *UGT2B7* is involved in oestrogen metabolism regulation, with a protective role from genotoxic oxidative products, which could alter the risk of developing cancer [26].

## 4. Discussion

**Figure 8:**
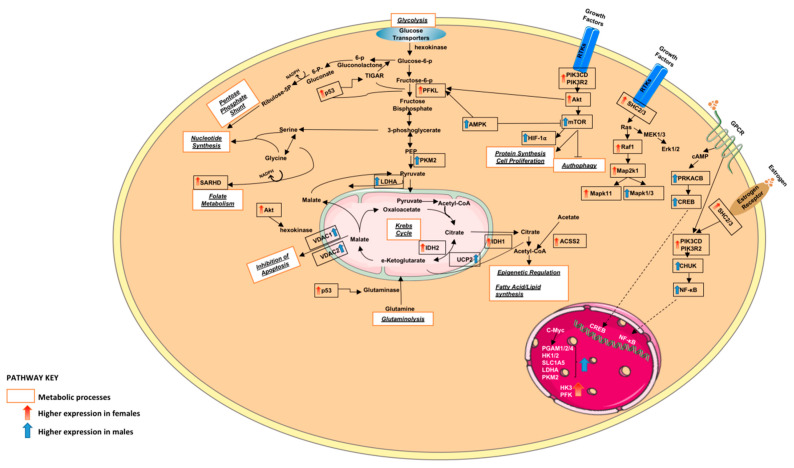
A graphical summary of some DEGs along with the metabolic processes they are involved in. Red denotes DEGs with elevated expression in females and blue denotes DEGs with elevated expression in males.

Circulating sex hormone actions do not account for all sex differences in cancer as sexual differentiation in normal physiology affects male and female rate of growth, immunity, myelination, aging and metabolism, amongst other physiological changes [27]. Studies demonstrate female LUAD patients having improved efficacy outcomes and longer survival regardless of therapeutic modality, stage, histological subtype and smoking status [28]. The results of this study show that males and females seem to favour different metabolic intermediates for energy production to support tumorigenesis (Figure 8), possibly allowing to identify and define the roles of critical molecular players/metabolites related to lung tumour formation.

The fundamental nutrient utilisation for metabolism in normal physiology differs between the sexes. In healthy humans, carbohydrate metabolites pathways, including glycolysis, glucogenesis and pyruvate, as well as amino acid metabolites, seem to be enriched in male serum compared to that of females. Furthermore, female embryos seem to favour the pentose phosphate pathway (PPP) and males glycolytic and higher glucose uptake. Additionally, it is reported that females seem to favour fatty acid metabolism [29]. Metabolic rewiring is an established cancer hallmark, best characterized by the “Warburg effect”, with cancer cells having more active glycolysis and defective mitochondrial ATP, resulting in reduced cellular NADPH/NADP+ [30]. Our female LUAD cohort seemed to exhibit the Warburg phenomenon, favouring glycolytic metabolism, glucogenesis and fatty acid/lipid synthesis, whereas the males used OXOPHOS and pyruvate metabolism. Furthermore, in our male cohort, much of the pyruvate from glycolysis appears to be directed away from the mitochondria through action of upregulated lactate dehydrogenase (*LDHA*) to create lactate, which is usually reserved for low oxygen state/aerobic glycolysis.

TP53-induced glycolysis and apoptosis regulator (TIGAR) play a regulatory role in cancer energy metabolism and cause increased NADPH production by PPP [31]. Through oxidative PPP, malic enzyme IDH1, its gene also upregulated with higher expression in our male cohort, generates NADPH-reducing equivalents, which are part of the defence against increased ROS. This could be of importance since TIGAR acts to reduce fructose-2,6-bisphosphate levels (Fru-2,6-P2) and upregulate glucose-6-phosphate dehydrogenase (G6PD) activity, which is a rate-limiting enzyme vital for DNA repair and nucleotide synthesis. A study by Liu et al. reported that TIGAR and SCO2 correlate with LUAD development, metastasis and higher mortality rate. They also confirmed our observation that glucose metabolism seems to play a regulatory role in LUAD. They also found TIGAR protein to be expressed to a greater extent in LUAD than in normal lung tissue, and that it associates with male LUAD samples [31].

We also identified sex-associated differences in tumour basic energy metabolism, which further highlights signalling cross-talk by nutrient sensing. Nutrient sensing is a crucial part of mTOR and AMPK pathways, affecting their interaction with one another, but it also creates metabolic flexibility to maintain homeostasis [32]. Similarly, the PI3K/AKT/mTOR signalling pathway regulates proliferation, survival and angiogenesis through promoting glycolysis, enhanced cellular AA consumption, lipid biogenesis and reducing cellular autophagy [33]. We identified *AMPK*, which is upregulated with higher expression in our male cohort to decrease expression of gluconeogenic enzymes, whereas, in our female cohort, the AMPK pathway is attenuated by higher expression of mTOR signalling pathway genes, directly phosphorylating *PPARGC1A* and resulting in activated mitochondrial biogenesis. Additionally, we observed in our female cohort, following PIK3 activation, that AKT exerts an antagonistic effect to regulate mTOR, promoting glucose transporter activity and stimulating glycolysis through activation of glycolytic enzymes, including hexokinase and phosphofructokinase. This results in overexpression of *HK1*, *PGAM2*, *PFKL* and of *GLS*, causing enhancement in glutamate metabolism. We propose that shifting of the cell’s metabolic programme from catabolic to anabolic, promoting aerobic glycolysis, is due to its MAPK pathway being amplified in our female cohort.

In addition to the widespread sex-associated differences between females and males are differences in drug metabolism and/or sensitivity, susceptibility and survival. Regarding LUAD, data clearly demonstrate that females are more susceptible to toxicity of different types of drugs. Xenobiotic metabolism generates activated metabolites that alter humoral and cellular immune response [34]. The AHR pathway is a sensor and regulator of the defence system against xenobiotic chemicals and is also linked with TGF-β signalling to downregulate SMAD4 and impair invasive capacity and activate autophagy in lung cancers [35]. The upregulated genes with higher expression in our male cohort involved in xenobiotic metabolism and part of ARH signalling include the GSTs: *GSTM4* and *GSTA2*. GSTs and UGTs are enzymes involved in detoxification of glutathione-S-transferases and UDP-glucuronosyltransferases, respectively, and play a crucial role in drug metabolism [36]. Interestingly, in our male cohort, regardless of smoking status, both MN and MC had higher expression levels of *GSTA2* and *GSTO2* (log_2_FC > 1.2) compared to females, whereas, in non-smokers, only females seemed to have a higher expression level of *UGT1A9* (log_2_FC > 1.2) (Supplementary Figure S6). This could also indicate that, in our LUAD cohort, the defence system in males regardless of smoking status seems to be constantly active, whereas, in female non-smokers, it seems to be more active compared to smokers and possibly one of the several reasons for their different response to therapy.

Immune responses show significant distinctions between females and males, indicating multiple potential differences in the biology of cancers arising in both sexes [37]. Indeed, our results also indicate immune responses to be heightened in our female LUAD cohort. We found the expression levels of *HLA-DQB1* to be significantly correlated with the levels of infiltrating B cells, macrophages, CD8+ T cells, CD4+ T cells, neutrophils, dendritic cells and Tregs in females. Its expression in LUAD tumour cells was found to be a prognostic marker of overall survival (OS) and to be associated with anti-tumour immunity [38]. Furthermore, sex differences in APC (Supplementary Figure S2) have a significant impact on anti-tumour immunity and immunotherapy response. APCs contribute to Tregs function and have been found to be regulated in an oestrogen-dependent manner. Moreover, females have higher macrophages and neutrophils activity than males, possibly indicative of why males are more susceptible to developing cancer. Immune cells infiltrated in the TME play key roles in tumorigenesis and progression [39] and are critical discriminants of tumour stratification and prognosis, correlating with LUAD’s development and progression [40]. Being a master regulator of cellular metabolism dysregulation of the P13K/Akt/mTOR signalling pathway contributes to several pathological conditions, including tumour progression, maintenance and metastasis. Sensing and integrating inputs from a variety of environmental signals to regulate immune cell trafficking in the TME promotes progression and metastasis and also determines and dictates immune cell fate decisions in T effector, memory and Treg cells [41].

Tumour cells in the TME outcompete T cells for glucose, which leads to sustained mTOR signalling, glycolysis and proliferation [41], as observed in our female cohort. On the contrary, downregulation of mTOR by enhanced glucose consumption results in chemokine secretion, which impairs T cells’ metabolic reprogramming and facilitates tumour immune escape [41]. We found overexpression of *FCER2, FXYD5, HMHA1* and *RENBP* to correlate with better prognosis in males, whereas, in females, higher expression of *GIPR*, *PRG2* and *TNFAIP2* correlates with better prognosis. In our female cohort, through MAPK signalling, higher expression of *MAPK1/3* could result in anergy via *JUN*, which is reported to occur in T cells infiltrating tumours. A similar response occurs following overexpression of *RELA* and *MAPK11* and *NFATC1/2*. Additionally, following indirect *JUN* activation via *EGFR* or *MAPK1/3*, it could result in angiogenesis and immune response. *MAPK1/3* and *RELA* seem to be major players in inflammatory immune response in our female cohort following cytokine receptor interaction of *CX3CL1* with *CXCR5*. We speculate this chemokine signal could correlate with tumour-infiltrating lymphocytes as a cancer immune evasion mechanism against immunosurveillance following altered Warburg effect, whereby glucose consumption and oxidation are dysregulated, enabling rapid proliferation [30].

It is established that smoking carcinogens contribute to the incidence of lung cancer initiation or progression, yet around 20% of smokers acquire lung cancer, indicating that other factors predispose its development, including, sex, genetic alteration, diet, comorbidities and even second-hand exposure [42]. It is inconclusive as to whether females might metabolise smoke carcinogens differently from males. Two potential mechanisms might justify the results: either an imbalance between metabolic activation and detoxification of carcinogens or a defective DNA repair system [3]. Our data seem to indicate hormones do play a role and that their immediate reduced levels following smoking cessation might be indicative of hormone-related disease risks modifiable with a change in lifestyle to preserve the integrity of hormone receptors in males and females. In fact, (1) smoke exposure increased free oestradiol and testosterone levels in overweight females. (2) In postmenopausal women, cigarette smoking associates with higher levels of circulating testosterone, androgen and oestrogen. (3) Androstenedione levels also increased with smoke exposure, with increased levels in postmenopausal smokers compared to non-smokers. (4) A possible mechanism by which smoking might affect AR levels is by decreasing their metabolic clearance, where nicotine inhibits conversion of androgens to oestrogens by aromatase [43]. We identified variations in gene expression of some genes encoding carcinogen-metabolising enzyme, such as *GSTM3*, which had higher expression in males, and *EGFR*, with higher expression in females. The confounding effect of tobacco exposure could partially explain this difference as heavy smoking was found to overburden the defence mechanisms [44]. Our results indicate a difference in expression levels of ERα and ERβ and androgen transcription targets by sex, as well as smoking status, but mostly due to sex; thus, smoking may influence hormone receptor expression levels.

**Figure 9:**
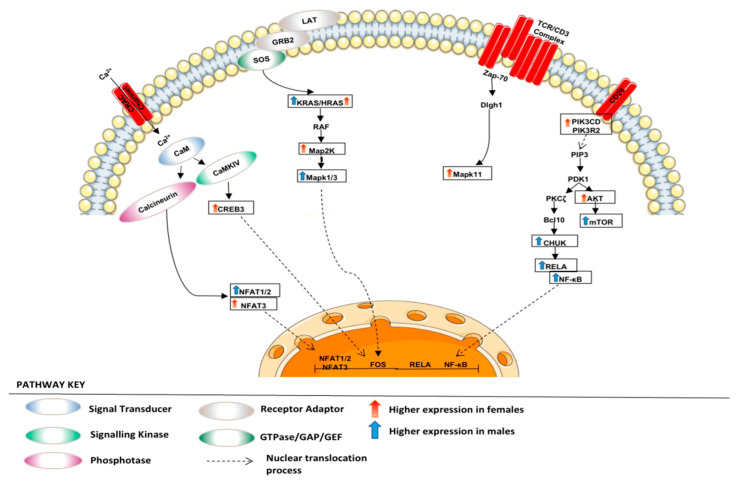
Regulation of T cell receptor signalling. The figure depicts various adaptors and enzymes involved in regulating TCR signalling in our LUAD cohort.

Interplay between steroid hormones and epigenetic reprogramming of cells is of great interest [8]. We identified *CALCR* to be upregulated in male DEGs, with completely contrasting expression profiles in males and females regardless of smoking status (Figure 9). We here propose a model for oestrogen protection. Evidence suggests that premenopausal women are protected against cardiovascular diseases, but postmenopausal women have the same risk as men since oestrogen seems to be responsible for the increased risk prevalence in women [45]. One of the DEGs we identified across the three groups testing the oestrogen protection hypothesis (pre-/postmenopausal females and males), *TIFF1*, seems to show a difference in sex rather than menopause status, possibly as it is expressed in gastrointestinal mucosa and its expression could be influenced by the gut microbiome, further contributing to the speculation that factors including different dietary choices between the sexes might result in such differences. In fact, in NSCLC in vitro studies, oestrogen was found to modulate *EGFR* levels and *EGF* to modulate ER levels. *EGFR* protein expression is downregulated in response to oestrogen and upregulated in response to anti-oestrogens, demonstrating crosstalk between ER and EGFR pathways [4]. In colon cancer, *EGFR* has a positive association with female survival compared to a negative one in males, suggesting interplay between *EGFR* and ERβ between the sexes [8]. This is interesting indeed as *EGFR* was only differentially expressed in our female cohort. As steroid hormones are central regulators of systemic metabolism, ERs and ARs increase glucose tolerance and restrain visceral fat accumulation, and, thus, in postmenopausal women with reduced ERα signalling and with individuals with aromatase deficiency having compromised AR to ER conversion, tend to have increased adiposity, which is a risk factor for cancer development [8]. Leptin receptor *LEPROTL1*, which indicates activity of leptin in our cohort, appeared to have higher expression in males compared to females; however, we were not able to investigate it further due to missing clinical information regarding weight.

There are limitations to this study. First, we were not able to fully exclude the effect of tobacco smoke in examining sex differences due to the small number of non-smokers (*n* = 75 in total). However, we previously investigated the global gene expression in LUAD tumour samples of smokers and non-smokers and highlighted the effect of cigarette smoking on tumour differentiation [9]. Second, females’ reproductive history may be prone to misclassification as we chose 55 as the age of menopause since information regarding the matter was lacking. Additionally, we had no information on use of oral contraceptives or hormonal replacement therapy and lacking this information may have affected our assessment of the association between sex hormones and LUAD progression. Despite these limitations, we believe we have provided a genome-wide overview of the drivers of differences in LUAD based on sex that exist in core cancer pathways. We aim to make the case for sex inclusion as a biological variable in evaluation of potential disease management, biomarker development and future therapeutic targets. Future stratification by these molecular markers could be valuable to uncover the link between LUAD sex differences in metabolism and molecular biology.

## 5. Conclusions

Several genes’ expression changes accompany lung cancer, attributed to carcinogenesis exposure, genetic, biological, demographic, environmental and even behavioural factors. The scale and variety of these factors have made it difficult to discriminate central processes and their relationship with prognosis. In the present study, through differential gene analysis, we were able to identify differentially expressed genes in both phenotypes. We identified dissimilarities in immune response and cellular homeostasis between the phenotypes, as well as different signalling pathways and metabolic processes. We aimed to investigate whether smoking or the absence of smoking affect regulation of oestrogen and androgen transcriptional targets in females and males. Additionally, we employed males, pre- and postmenopausal females to assess oestrogen protection in the compared groups. There are already several papers published about environmental factors affecting cancer susceptibility; however, regarding differences between males and females, no strong evidence is known about lung cancer. We know that diet and hormonal status affect cancers such as colorectal cancer or hormonal-dependent tissue cancer (BRCA and PRAD). In our present study, we also showed that, in lung cancer, environmental factors including diet present as a direct association with cancer progression and prognosis and need to be taken into account.

## Figures and Tables

**Table 1:** Enrichment analysis regarding sex-disparate pathways following DEG identification and confirmation using EdgeR (*p* < 0.05). This table details a selection of pathways significantly enriched in our LUAD cohort using ShinyGo enrichment analyser (Supplementary Tables S4 and S5) and following literature search with validation from Human MSigDB gene collection. Red denotes DEGs with elevated expression in females and blue denotes DEGs with elevated expression in males.

**Cell Cycle**	*STK11*, *BIRC7*, *CROCC*, *AKR1B10*, *C19orf21*, *MST4*, *PTP4A1*
**Proliferation**	*AKR1C2*, *AKR1C3*, *ID1*, *IRS2*, *PPARGC1A*, *PTHLH*, *TFF1*, *AKR1B10*, *FOXP2*, *APOE*, *BOK*, *CBFA2T3*, *CD79A*, *CX3CL1*, *ENG*, *MAPK11*, *MS4A1*, *STK11*, *TNFRSF13B*, *TNFSF14*, *ICOSLG*, *MATK*
**Apoptosis**	*BOK*, *STK11*, *CD14*, *PPARGC1A*, *AKR1E2*, *ATP1B1*, *MST4*, *SLCO1A2*
**PI3K-AKT Signalling Pathway**	*CDKN1A*, *EGFR*, *MTOR*, *PIK3CD*, *PIK3R2*, *CDKN1B*, *CHUK*, *IRS2*, *KIT*
**MAPK signalling pathway**	*MAP2K1*, *PRKACB*, *CHUK*, *DUSP4*, *KRAS*, *MAPK11*, *CD14*, *TGFB1*, *EGFR*, *MAPK1*, *MAPK3*, *NFATC2*, *RELA*, *TGFB2*, *GADD45A*, *TGFBR2*, *CACNA1I*
**ATP-dependent transporter of the ATP-binding cassette (ABC)**	*MGST1*, *AKR1C3*, *ALDH3B1*, *TAT*, *AKR1C1*, *AKR1C2*, *ITIH4*, *IGFBP1*, *GSR*, *ABCC2*, *PGD*, *ENPEP*, *MCCC2*, *GABARAPL1*, *GSTM4*, *GSTO2*, *GSTA2*, *PTGES3*, *AHCY*, *CYP2C8*, *CBR1*, *APOE*, *PINK1*, *ALDH1A3*, *GSTA3*, *PPARD*, *TGFB2*
**HIF PID signalling pathway**	*ENG*, *GATA2*, *FURIN*, *IGFBP1*
**FOXO signalling pathway**	*ARG1*, *APOE*, *ATM*, *CDKN1A*, *EGFR*, *GABARAP*, *GADD45A*, *MAPK11*, *MAPK1*, *MAPK3*, *PIK3CD*, *PIK3R2*, *TGFB1*, *TGFB2*, *TGFBR2*, *STK11*, *SREBF1*, *CCNB3*, *CCND1*, *CDKN1B*, *CHUK*, *GABARAPL1*, *INSR*, *IRS2*, *KRAS*, *MAP2K1*, *PRKAB2*, *PRKAG2*, *PRMT1*, *SIRT1*, *SMAD4*, *EIF4E*, *ULK2*, *FOXP2*, *PPARGC1A*
**AMPK signalling pathway**	*CCND1*, *GYS2*, *INSR*, *IRS2*, *LEP*, *PPARGC1A*, *PPP2CB*, *PPP2R1A*, *PPP2R1B*, *PPP2R5C*, *PRKAB2*, *PRKAG2*, *SIRT1*, *SREBF1*, *STK11*, *CCNA1*, *PPP2R5B*, *CREB3*, *PFKL*, *PIK3CD*, *PIK3R2*, *RAB8A*, *STK11*, *STRADB*, *TBC1D1*
**mTOR signalling pathway**	*EIF4B*, *INSR*, *SEH1L*, *CHUK*, *MAP2K1*, *ATP6V1G1*, *EIF4E*, *ULK2*, *PTHLH*, *STK11*, *MAPK1*, *PIK3R2*, *WNT6*, *ATP6V1E1*, *FZD7*, *STK11*, *TELO2*, *MAPK3*, *WNT5B*, *FZD2*, *MTOR*, *FLCN*, *PIK3CD*, *WDR24*, *NPRL2*, *STRADB*, *PIK3CG*
**TIMER2.0 immune related genes**	*RENBP*, *ARHGAP22*, *CD79A*, *CXCR5*, *FCER2*, *GIPR*, *MS4A1*, *NCR3*, *PRG2*, *RYR1*, *SLC15A3*, *TNFRSF13B*, *TNFSF14*, *VPREB3*, *FGD3*, *FXYD5*, *HLA-DQB1*, *HMHA1*, *ICOSLG*, *TMC8*, *TNFAIP2*, *HAL*
**Warburg Effect**	*ACSS1*, *PFKL*, *PIK3CD*, *GLS*, *EGFR*, *HK1*, *MAPK1*, *PGAM2*, *MAPK3*, *PIK3R2*, *PDHB*, *KIT*, *KRAS*, *G6PD*, *MAP2K1S*
**ARH signalling pathway**	*CDKN1A*, *EGFR*, *RELA*, *TGFB1*, *MAPK1*, *GSTA2*, *IGFBP1*, *KRAS*, *MAP2K1*, *CDKN1B*
**Metabolism of xenobiotics by cytochrome P450**	*CYP2C8*, *MGST1*, *AKR1C3*, *ALDH3B1*, *AKR1C2*, *AKR1C1*, *GSTM4*, *GSTO2*, *GSTA2*, *ALDH1A3*, *CYP2S1*, *GSTA3*
**RAS signalling pathway**	*APOE*, *MAPK11*, *RTN4R*, *SHC3*, *STMN3*, *TIAM1*, *SHC2*
**Wnt signalling pathway**	*NFATC3*, *WNT6*, *FZD7*, *TBL1Y*, *NFATC2*, *CCND1*, *WNT5B*, *PRKACB*, *FZD2*
**Reactive Oxygen Species pathway**	*ABCC1*, *G6PD*, *GLRX2*, *GSR*, *MGST1*, *NDUFB4*, *PRDX4*, *PRDX6*, *TXNRD2*

**Table 2:** Correlation analysis between candidate hub genes and immune cells from TIMER2.0 database. Red denotes DEGs with elevated expression in females and blue denotes DEGs with elevated expression in males.

DEGs	Purity		B Lymphocytes		CD8+ T cell		CD4+ T cell		Macrophages		NEUTROPHILES		DC		Tregs	
	R^2^	P	R^2^	P	R^2^	P	R^2^	P	R^2^	P	R^2^	P	R^2^	P	R^2^	P
*ARHGAP22*	−0.229	2.65 × 10^−7^	0.175	9.26 × 10^−5^	0.063	1.63 × 10^−1^	0−0.204	5.07 × 10^−6^	0.1	2.65 × 10^−2^	0.045	3.22 × 10^−1^	0.141	1.66 × 10^−3^	0.189	2.37 × 10^−5^
*CD79A*	−0.457	7.17 × 10^−27^	0.718	1.89 × 10^−79^	0.403	1.06 × 10^−20^	−0.161	3.36 × 10^−4^	−0.013	7.79 × 10^−1^	−0.142	1.56 × 10^−3^	0.071	1.15 × 10^−1^	0.482	4.26 × 10^−30^
*CXCR5*	−0.505	2.24 × 10^−33^	0.731	1.66 × 10^−83^	0.45	6.43 × 10^−26^	−0.115	1.03 × 10^−^2	0.135	2.69 × 10^−3^	−0.025	5.84 × 10^−1^	−0.004	9.27 × 10^−1^	0.501	1.04 × 10^−32^
*FCER2*	−0.424	5.88 × 10^−23^	0.58	1.33 × 10^−45^	0.271	1.01 × 10^−9^	−0.022	6.23 × 10^−1^	0.104	2.06 × 10^−2^	0.004	9.34 × 10^−1^	−0.073	1.06 × 10^−1^	0.376	5.14 × 10^−18^
*FGD3*	−0.423	7.46 × 10^−23^	0.455	1.58 × 10^−26^	0.498	3.08 × 10^−32^	−0.215	1.52 × 10^−6^	0.282	1.72 × 10^−10^	0.02	6.65 × 10^−1^	0.009	8.40 × 10^−1^	0.516	7.64 × 10^−35^
*FXYD5*	−0.267	1.60 × 10^−9^	0.031	4.89 × 10^−1^	0.06	1.83 × 10^−1^	−0.026	5.70 × 10^−1^	0.328	8.45 × 10^−14^	0.233	1.77 × 10^−7^	−0.198	9.33 × 10^−6^	0.069	1.28 × 10^−1^
*GIPR*	−0.104	2.12 × 10^−2^	0.21	2.61 × 10^−6^	0.044	3.20 × 10^−1^	−0.148	9.68 × 10^−4^	0.205	4.46 × 10^−6^	0.171	1.39 × 10^−4^	−0.079	7.89 × 10^−2^	0.362	1.09 × 10^−16^
*HAL*	0.047	2.90 × 10^−1^	−0.063	1.59 × 10^−1^	−0.158	4.23 × 10^−4^	0.129	4.04 × 10^−13^	−0.191	1.93 × 10^−5^	0.071	1.16 × 10^−1^	0.08	7.64 × 10^−2^	−0.122	6.48 × 10^−3^
*HLA−DQB1*	−0.353	6.30 × 10^−16^	0.247	2.90 × 10^−8^	0.226	3.87 × 10^−7^	−0.126	4.99 × 10^−3^	0.445	2.51 × 10^−25^	0.1	2.70 × 10^−2^	−0.204	4.82 × 10^−6^	0.342	5.78 × 10^−15^
*HMHA1*	−0.277	3.97 × 10^−10^	0.218	1.06 × 10^−6^	0.21	2.54 × 10^−6^	−0.234	1.39 × 10^−7^	0.359	2.02 × 10^−16^	0.063	1.65 × 10^−1^	−0.056	2.13 × 10^−1^	0.408	3.49 × 10^−21^
*ICOSLG*	−0.233	1.59 × 10^−7^	0.3	1.06 × 10^−11^	0.12	7.55 × 10^−3^	−0.042	3.49 × 10^−1^	0.181	5.51 × 10^−5^	0.038	3.97 × 10^−1^	0.023	6.11 × 10^−1^	0.401	1.80 × 10^−20^
*MS4A1*	−0.502	7.29 × 10^−33^	0.74	1.19 × 10^−86^	0.441	7.14 × 10^−25^	−0.086	5.76 × 10^−2^	0.082	6.93 × 10^−2^	−0.068	1.30 × 10^−1^	−0.028	5.32 × 10^−1^	0.454	2.22 × 10^−26^
*NCR3*	−0.503	4.88 × 10^−33^	0.607	5.62 × 10^−51^	0.63	5.46 × 10^−56^	−0.101	2.46 × 10^−2^	0.155	5.68 × 10^−4^	−0.092	4.01 × 10^−2^	−0.079	7.94 × 10^−2^	0.431	9.64 × 10^−24^
*PRG2*	−0.122	6.43 × 10^−3^	−0.022	6.32 × 10^−1^	−0.061	1.76 × 10^−1^	−0.236	1.11 × 10^−7^	0.25	1.95 × 10^−8^	0.197	1.06 × 10^−5^	−0.085	5.97 × 10^−2^	0.179	6.57 × 10^−5^
*RENBP*	−0.237	9.51 × 10^−8^	0.173	1.18 × 10^−4^	0.236	1.16 × 10^−7^	−0.178	7.16 × 10^−5^	0.268	1.42 × 10^−9^	0.071	1.16 × 10^−1^	0.01	8.28 × 10^−1^	0.265	2.28 × 10^−9^
*RYR* *1*	−0.112	1.31 × 10^−2^	0.05	2.63 × 10^−1^	0.092	4.18 × 10^−2^	−0.113	1.20 × 10^−2^	0.341	6.33 × 10^−15^	0.234	1.47 × 10^−7^	−0.094	3.68 × 10^−2^	0.203	5.50 × 10^−6^
*SLC15A* *3*	−0.431	9.65 × 10^−24^	0.269	1.23 × 10^−9^	0.345	2.90 × 10^−15^	−0.132	3.35 × 10^−3^	0.383	1.16 × 10^−18^	0.071	1.18 × 10^−1^	−0.107	1.76 × 10^−2^	0.461	2.38 × 10^−27^
*TMC* *8*	−0.432	8.18 × 10^−23^	0.404	8.79 × 10^−21^	0.406	5.60 × 10^−21^	−0.169	1.68 × 10^−4^	0.344	4.07 × 10^−15^	0.071	1.18 × 10^−1^	−0.19	2.10 × 10^−5^	0.472	1.17 × 10^−28^
*TNFAIP* *2*	−0.311	1.51 × 10^−12^	0.167	2.01 × 10^−4^	0.3	1.03 × 10^−11^	−0.19	2.25 × 10^−5^	0.312	1.44 × 10^−12^	0.041	3.59 × 10^−1^	−0.029	5.26 × 10^−1^	0.275	5.45 × 10^−10^
*TNFRSF13B*	−0.438	1.37 × 10^−24^	0.717	3.98 × 10^−79^	0.365	5.76 × 10^−17^	−0.096	3.23 × 10^−2^	0.041	3.69 × 10^−1^	−0.039	3.84 × 10^−1^	−0.066	1.46 × 10^−1^	0.478	1.79 × 10^−29^
*TNFSF14*	−0.356	3.34 × 10^−16^	0.21	2.67 × 10^−6^	0.206	4.18 × 10^−6^	0.292	3.95 × 10^−11^	0.181	6.01 × 10^−5^	0.045	3.22 × 10^−1^	−0.109	1.52 × 10^−2^	0.264	2.70 × 10^−9^
*VPREB* *3*	−0.442	5.33 × 10^−25^	0.688	2.18 × 10^−70^	0.308	2.53 × 10^−12^	−0.116	1.01 × 10^−2^	−0.042	3.47 × 10^−1^	−0.132	3.26 × 10^−3^	0.042	3.50 × 10^−1^	0.284	1.34 × 10^−10^

## Data Availability

LUAD TCGA RNA-seq data are publicly available from (http://gdac.broadinstitute.org/ data status of 28 January 2016). For validation, gene chip GSE12667 of LUAD with its clinical manifestation data was also downloaded from the Genome Expression Omnibus (GEO) database, available from https://www.ncbi.nlm.nih.gov/geo/ accessed on 21 March 2021. Supplementary data from our analyses are also available in the Supplementary Materials.

## Supplementary Materials

The following supporting information can be downloaded at: https://www.mdpi.com/article/10.3390/curroncol30020107/s1, Table S1: LUAD patient clinical information; Table S2: List of DEGs with log fold change and p-value using EdgeR; Tabled S3: Associated GO terms for 9 DEGs upregulated in males; Table S4: Enrichment analysis using ShinyGO ranked by fold enrichment; Table S5: GSEA Enrichment analysis; Figure S1: MD plot showing the log-fold change and average abundance of each gene. Significantly up and down DEGs are highlighted in red and blue, respectively; Figure S2: “Control” genes encoded on the sex chromosomes in LUAD cohort showing no difference in expression between the two phenotypes, thus confirming specificity of our DEGs in regard to LUAD rather than a general sex-specific difference; Figure S3: Expression of ATP-binding cassette (ABC) transporters in LUAD patient groups according to smoking status and sex; Figure S4: Correlation of DEGs mRNA expression with immune cell infiltrates from TIMER 2.0 in LUAD patient groups according to smoking status and sex; Figure S5: Expression of immune related DEGs in LUAD patient groups according to smoking status and sex; Figure S6: Expression of detoxification related genes s in LUAD patient groups according to smoking status and sex.
