# Supplementary material for: X or Y Cancer: An Extensive Analysis of Sex Differences in Lung Adenocarcinoma
Source: Curr Oncol. 2023 Jan 18;30(2):1395–415. doi: 10.3390/curroncol30020107 (PMC9955992; doi:10.3390/curroncol30020107)
Supplement: Supplementary file 1 [file curroncol-30-00107-s001.zip › curroncol-2173650-supplementary.pdf]

Supplementary Data

X Or Y Cancer: An Extensive Analysis of Sex Differences in Lung Adenocarcinoma

Raneem Yaseen Hammouz *, Magdalena Orzechowska, Dorota Anusewicz and Andrzej K. Bednarek

Department of Molecular Carcinogenesis, Medical University of Lodz, 90-752 Lodz, Poland

* Correspondence: raneem.hamouz@umed.lodz.pl

Supplementary Table S1. LUAD patient cohort clinical information.

Parameter	Males	Females
**Quantity**	229	268
**Median age(range)**	66 (38-88)	66 (39-87)
**Stage**		
**I**	111(48.4)	158(59)
**II**	64(27.9)	51(19)
**III**	35(15.3)	45(16.8)
**IV**	14(6.1)	12(4.4)
**NA**	5	2
**alive**	177	205
**dead**	52	63
**current smokers**	69	51
**never smokers**	20	55
**reformed smokers**	140	162

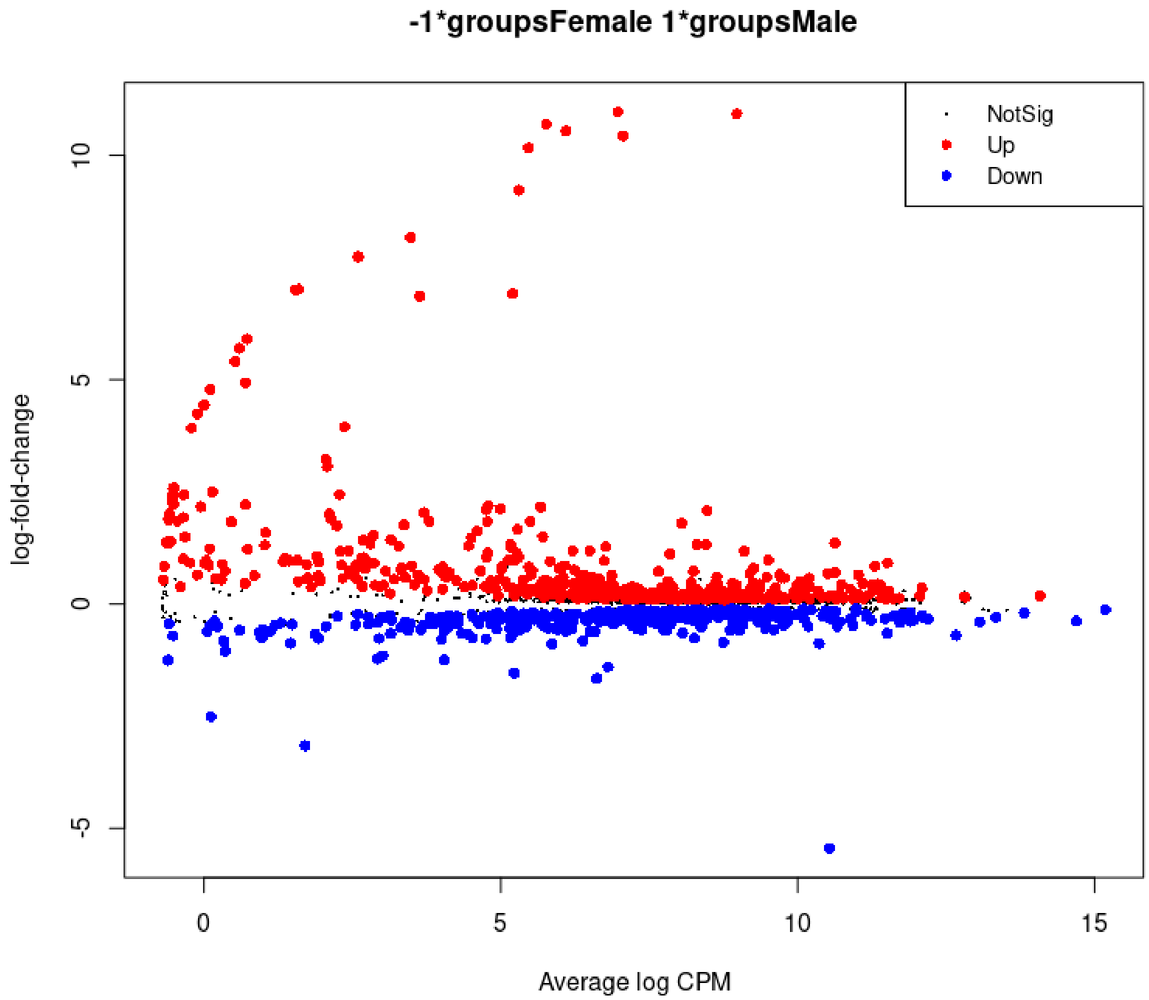

Supplementary Figure S1. MD plot showing the log-fold change and average abundance of each gene. Significantly up and down DEGs are highlighted in red and blue, respectively.

Supplementary table S2. List of DEGs with log fold change and p-value using EdgeR.

Gene name	logFC	PValue
ABCC1	0.0357	0.0358
ABCC2	1.3643	0.0000
ACSS1	-0.0448	0.0387
ACSS2	-0.1097	0.0146
AHCY	0.1590	0.0066
AKR1B10	0.8525	0.0067
AKR1C1	0.9467	0.0001
AKR1C2	0.8767	0.0010
AKR1C3	0.6210	0.0009
AKR1E2	0.3466	0.0083
ALDH1A3	-0.2751	0.0236
ALDH3B1	0.3625	0.0007
APOE	-0.1627	0.0340
ARG1	1.3661	0.0000
ARHGAP22	0.0618	0.0370
ATM	-0.0744	0.0458
ATP12A	-0.5403	0.0010
ATP1B1	0.3706	0.0001
ATP6V0A4	0.3863	0.0496
ATP6V1E1	-0.0736	0.0471
ATP6V1G1	0.0593	0.0269
BIRC7	-0.2509	0.0038
BOK	-0.1611	0.0430
C19orf21	0.3570	0.0006
CACNA1I	-0.6821	0.0001
CBFA2T3	-0.3165	0.0055
CBR1	0.6957	0.0000

CCNA1	-0.0075	0.0264
CCNB3	0.3131	0.0135
CCND1	0.0045	0.0235
CD14	-0.0248	0.0316
CD79A	-0.2636	0.0491
CDKN1A	-0.0974	0.0352
CDKN1B	0.0531	0.0267
CHUK	0.1866	0.0000
CLDN9	-0.8941	0.0000
CREB3	-0.0309	0.0336
CROCC	-0.1384	0.0037
CX3CL1	-0.5643	0.0000
CXCR5	-0.6396	0.0000
CYP2C8	0.3725	0.0239
CYP2S1	0.2123	0.0155
DDX3Y	8.8041	0.0000
DUSP4	0.5227	0.0003
EGFR	-0.5494	0.0000
EIF1AY	8.9668	0.0000
EIF4B	0.0895	0.0492
EIF4E	0.1192	0.0060
ENG	-0.3003	0.0000
ENPEP	0.0444	0.0418
FCER2	-1.1476	0.0000
FGD3	-0.2078	0.0136
FLCN	-0.0338	0.0346
FOXP2	0.5004	0.0002
FURIN	0.4790	0.0000

FXYD5	-0.2379	0.0049
FZD2	-0.3668	0.0002
FZD7	-0.1074	0.0104
G6PD	0.4414	0.0003
GABARAP	0.2555	0.0008
GABARAPL1	0.0095	0.0165
GADD45A	-0.0446	0.0387
GATA2	-0.1414	0.0038
GIPR	-0.2298	0.0033
GLRX2	0.1456	0.0071
GLS	-0.2375	0.0301
GSR	0.5047	0.0000
GSTA2	0.9936	0.0001
GSTA3	0.3675	0.0001
GSTM4	0.1063	0.0048
GSTO2	0.5355	0.0000
GULP1	0.3796	0.0004
GYS2	0.7819	0.0004
HAL	0.5326	0.0048
HK1	-0.0225	0.0271
HLA-DQB1	-0.4721	0.0001
HMHA1	-0.1730	0.0158
HRAS	0.0847	0.0355
HSD17B2	0.6875	0.0029
ICOSLG	-0.1880	0.0057
ID1	0.4313	0.0003
IDH2	0.1841	0.0147
IGFBP1	2.2568	0.0000

IL4I1	-0.2118	0.0462
INSR	0.0205	0.0458
IRS2	0.3391	0.0031
ITIH4	0.0730	0.0365
KDM5D	9.8768	0.0000
KIT	0.3309	0.0269
KRAS	0.0187	0.0482
L1CAM	-1.5497	0.0000
LDHA	0.0462	0.0430
LEP	-0.3016	0.0260
MAP2K1	-0.0239	0.0298
MAPK1	-0.1855	0.0329
MAPK11	-0.0471	0.0398
MAPK3	-0.0233	0.0284
MCCC2	0.1884	0.0001
MGST1	0.2435	0.0063
MS4A1	-0.6055	0.0005
MST4	0.1933	0.0105
MTOR	-0.0029	0.0258
MYH2	-0.8591	0.0000
NCR3	-0.4250	0.0005
NDUFB4	0.0429	0.0462
NDUFV2	0.1428	0.0099
NFATC2	-0.2244	0.0428
NFATC3	-0.0375	0.0369
NLGN4Y	6.6386	0.0000
NOS1	1.1134	0.0000
NOX1	-1.1733	0.0000

NPRL2	-0.0078	0.0269
PDE4D	0.4587	0.0001
PDHB	0.0902	0.0138
PFKL	-0.0992	0.0444
PGAM2	-0.1020	0.0258
PGD	0.4828	0.0000
PIK3CD	-0.2910	0.0001
PIK3CG	-0.1976	0.0466
PIK3R2	-0.0477	0.0487
PINK1	-0.1206	0.0109
PPARD	-0.1176	0.0368
PPARGC1A	0.4587	0.0099
PPP2CB	0.0909	0.0453
PPP2R1A	0.0740	0.0335
PPP2R1B	0.3046	0.0000
PPP2R5B	-0.1605	0.0040
PPP2R5C	0.0117	0.0458
PRDX4	0.1763	0.0120
PRDX6	0.1359	0.0045
PRG2	0.0728	0.0491
PRKAB2	0.1248	0.0449
PRKACB	0.2970	0.0004
PRKAG2	0.0689	0.0359
PRKY	6.8290	0.0000
PRMT1	0.1364	0.0033
PTGES3	0.0483	0.0433
PTHLH	0.2619	0.0004
PTP4A1	0.2540	0.0011

RAB8A	-0.0872	0.0121
RELA	-0.0342	0.0351
RENBP	-0.3572	0.0003
RTN4R	-0.4723	0.0000
RYR1	-0.3920	0.0127
SCG3	1.9948	0.0000
SEH1L	0.1507	0.0045
SERPIND1	-1.3912	0.0000
SHC2	-0.1336	0.0255
SHC3	-0.4322	0.0015
SIRT1	0.0172	0.0325
SLC15A3	-0.2530	0.0031
SLCO1A2	1.3148	0.0000
SMAD4	0.0981	0.0293
SREBF1	0.0483	0.0393
STK11	-0.2364	0.0003
STMN3	-0.1383	0.0036
STRADB	0.0084	0.0216
SULT2B1	0.4543	0.0006
TAT	-0.0484	0.0306
TBC1D1	0.0901	0.0246
TBL1Y	5.3918	0.0000
TELO2	-0.0434	0.0380
TFF1	1.3582	0.0001
TGFB1	-0.1981	0.0042
TGFB2	-0.2078	0.0379
TGFBR2	-0.0812	0.0259
TIAM1	-0.3576	0.0004

TMC8	-0.3593	0.0000
TMSB4Y	7.4133	0.0000
TNFAIP2	-0.2215	0.0393
TNFRSF13B	-0.5151	0.0012
TNFSF14	-0.0445	0.0398
TSPAN8	0.7129	0.0002
TXNRD2	0.0432	0.0255
UCP2	0.1599	0.0365
UGT2B7	1.5914	0.0000
ULK2	0.0443	0.0418
USH1C	1.3136	0.0000
USP9Y	9.9404	0.0000
UTY	9.2930	0.0000
VPREB3	-0.5510	0.0001
WDR24	-0.0849	0.0152
WNT5B	0.0733	0.0321
WNT6	0.5283	0.0014
XIST	-5.3808	0.0000
ZFY	8.4948	0.0000

Supplementary Table S3. Associated GO terms for 9 DEGs upregulated in males.

GENE	GO Term Name	GO Term Accession
USP9Y	ubiquitin-dependent protein catabolic process	GO:0006511
	thiol-dependent ubiquitin-specific protease activity	GO:0004843
	protein deubiquitination	GO:0016579
	cytoplasm	GO:0005737
	peptidase activity	GO:0008233
	cysteine-type peptidase activity	GO:0008234
	proteolysis	GO:0006508
	hydrolase activity	GO:0016787
	nucleus	GO:0005634
	cytosol	GO:0005829
	cell migration	GO:0016477
	cysteine-type endopeptidase activity	GO:0004197
	BMP signaling pathway	GO:0030509
	transforming growth factor beta receptor signaling pathway	GO:0007179
	co-SMAD binding	GO:0070410
	spermatogenesis	GO:0007283
EIF1AY	RNA binding	GO:0003723
	translation initiation factor activity	GO:0003743
	translational initiation	GO:0006413
	translation	GO:0006412
	protein binding	GO:0005515
NLGN4Y	membrane	GO:0016020
	integral component of plasma membrane	GO:0005887
	cell adhesion	GO:0007155
	neurexin family protein binding	GO:0042043
	synapse	GO:0045202
	synapse organization	GO:0050808
	integral component of membrane	GO:0016021

	plasma membrane	GO:0005886
	cell junction	GO:0030054
	presynapse	GO:0098793
	postsynaptic membrane	GO:0045211
	postsynaptic density membrane	GO:0098839
	chemical synaptic transmission	GO:0007268
	signaling receptor activity	GO:0038023
	cell surface	GO:0009986
	synaptic vesicle endocytosis	GO:0048488
	neuron cell-cell adhesion	GO:0007158
	scaffold protein binding	GO:0097110
	modulation of chemical synaptic transmission	GO:0050804
	cell adhesion molecule binding	GO:0050839
	learning	GO:0007612
	postsynaptic membrane assembly	GO:0097104
	presynaptic membrane assembly	GO:0097105
	social behavior	GO:0035176
	spanning component of membrane	GO:0089717
	vocalization behavior	GO:0071625
	presynapse assembly	GO:0099054
	symmetric, GABA-ergic, inhibitory synapse	GO:0098983
	asymmetric, glutamatergic, excitatory synapse	GO:0098985
KDM5D	DNA binding	GO:0003677
	metal ion binding	GO:0046872
	oxidoreductase activity	GO:0016491
	fibrillar center	GO:0001650
	chromatin organization	GO:0006325
	dioxygenase activity	GO:0051213
	histone demethylase activity	GO:0032452
	chromatin remodeling	GO:0006338
	histone demethylase activity (H3-trimethyl-K4 specific)	GO:0034647
	T cell antigen processing and presentation	GO:0002457
	histone H3-K4 demethylation, trimethyl-H3-K4-specific	GO:0034721

	nucleoplasm	GO:0005654
	androgen receptor binding	GO:0050681
	histone H3-K4 demethylation	GO:0034720
	regulation of androgen receptor signaling pathway	GO:0060765
	histone demethylase activity (H3-K4 specific)	GO:0032453
RPS4Y1	structural constituent of ribosome	GO:0003735
	ribosome	GO:0005840
	multicellular organism development	GO:0007275
	cytosolic small ribosomal subunit	GO:0022627
	rRNA binding	GO:0019843
	viral transcription	GO:0019083
	nuclear-transcribed mRNA catabolic process, nonsense-mediated decay	GO:0000184
	SRP-dependent cotranslational protein targeting to membrane	GO:0006614
	polysome	GO:0005844
ZFY	regulation of transcription, DNA-templated	GO:0006355
	nucleolus	GO:0005730
	regulation of transcription by RNA polymerase II	GO:0006357
	DNA-binding transcription factor activity, RNA polymerase II-specific	GO:0000981
	sequence-specific DNA binding	GO:0043565
DDX3Y	ATP binding	GO:0005524
	nucleic acid binding	GO:0003676
	helicase activity	GO:0004386
	nucleotide binding	GO:0000166
	RNA helicase activity	GO:0003724
	ATPase activity	GO:0016887
	cell differentiation	GO:0030154
	gamete generation	GO:0007276
	P granule	GO:0043186
UTY	RNA polymerase II cis-regulatory region sequence-specific DNA binding	GO:0000978
	regulation of gene expression	GO:0010468
	chromatin DNA binding	GO:0031490

	MLL3/4 complex	GO:0044666
	heart development	GO:0007507
	histone demethylase activity (H3-K27 specific)	GO:0071558
	histone H3-K27 demethylation	GO:0071557
TMSB4Y	actin monomer binding	GO:0003785
	actin filament organization	GO:0007015
	actin binding	GO:0003779
	cytoskeleton	GO:0005856
	regulation of cell migration	GO:0030334
	sequestering of actin monomers	GO:0042989
	regulation of actin polymerization or depolymerization	GO:0008064

Supplementary table S4. Enrichment analysis using ShinyGO ranked by fold enrichment.

Pathway	Enrichment FDR	Fold Enrichment
FoxO signaling pathway	1.97E-31	27.7277074070967
AMPK signaling pathway	1.31E-26	26.0943223443223
MTOR signaling pathway	7.51E-24	20.333238190381
HIF-1 signaling pathway	4.60E-15	18.3857243673757
Metabolism of xenobiotics by cytochrome P450	1.46E-09	16.9260469260469
Apoptosis	3.71E-08	10.2057794057794
PI3K-Akt signaling pathway	1.52E-17	9.5531756379214
Cell cycle	1.97E-07	9.94069422640851
MAPK signaling pathway	2.13E-12	8.5205950512073
Ras signaling pathway	2.62E-10	8.67551496122925
Wnt signaling pathway	2.64E-07	8.29988084204952
ABC transporters	0.064235862944841	5.56678876678877

Supplementary table S5. GSEA Enrichment analysis.

**NAME**	**NES**	**FDR q-val**
KEGG_CELL_CYCLE	-1.57	0.203
KEGG_METABOLISM_OF_XENOBIOTICS_BY_CYTOCHROME_P450	-2.05	0.044
MTOR_UP.N4.V1_UP	-1.44	0.205
MTOR_UP.N4.V1_UP	-1.44	0.205
MTOR_UP.V1_UP	-1.36	0.266
HALLMARK_REACTIVE_OXYGEN_SPECIES_PATHWAY	-1.48	0.185
HALLMARK_MTORC1_SIGNALING	-1.69	0.2
HOLLMANN_APOPTOSIS_VIA_CD40_UP	1.74	0.191
GSE40274_FOXP3_VS_FOXP3_AND_SATB1_TRANSDUCED_ACTIVATED_CD4_TCELL_DN	1.76	0.102

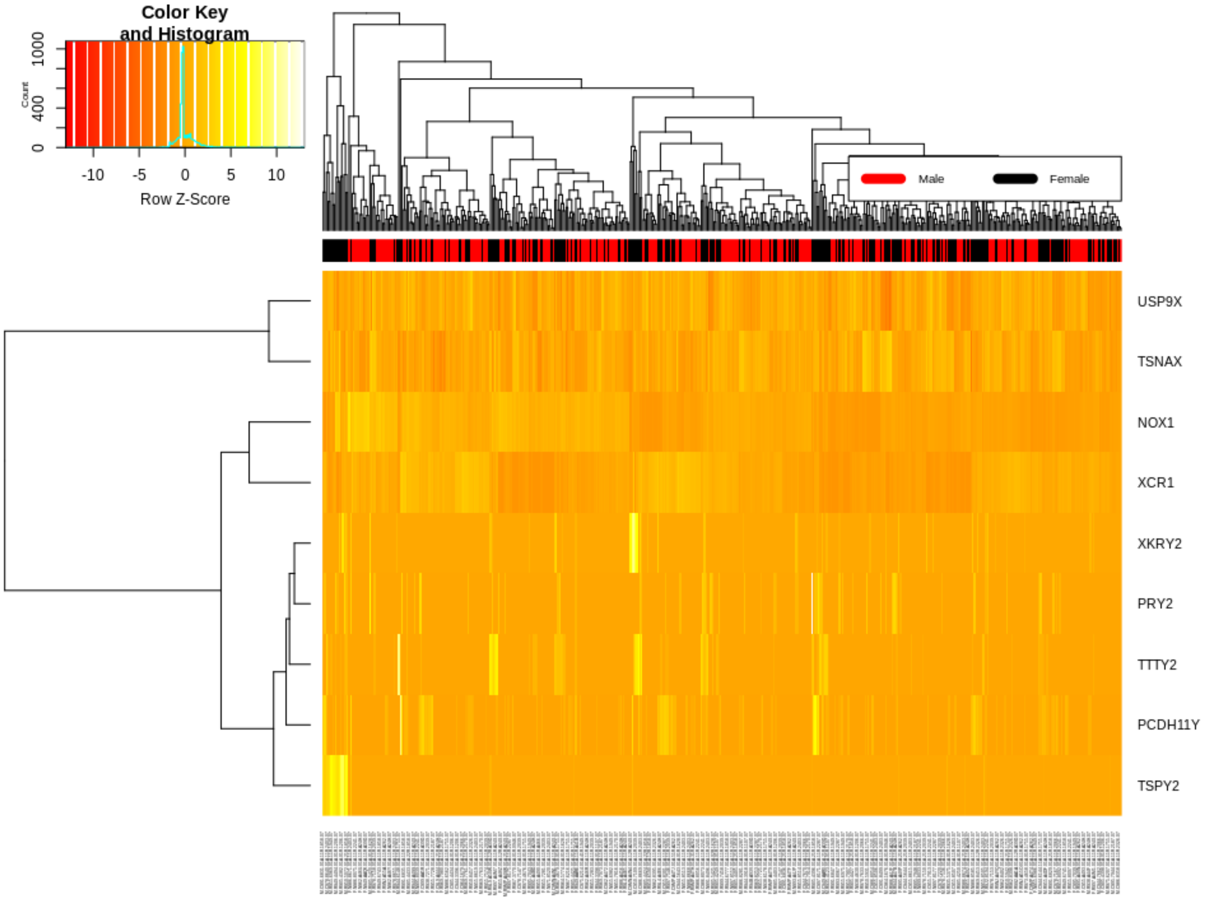

Supplementary Figure S2. “Control” genes encoded on the sex chromosomes in LUAD cohort showing no difference in expression between the two phenotypes, thus confirming specificity of our DEGs in regard to LUAD rather than a general sex-specific difference.

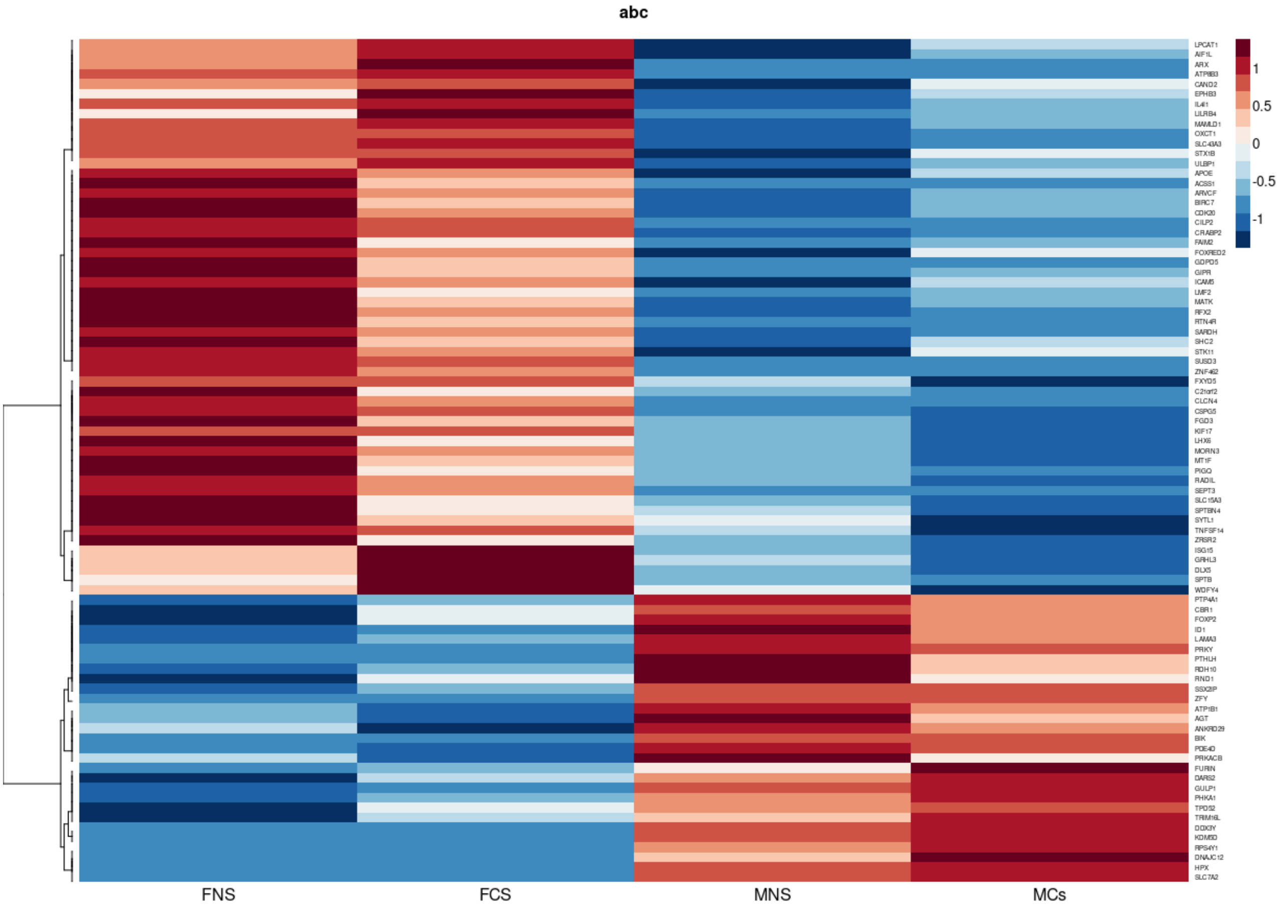

Supplementary Figure S3. Expression of ATP-binding cassette (ABC) transporters in LUAD patient groups according to smoking status and sex.

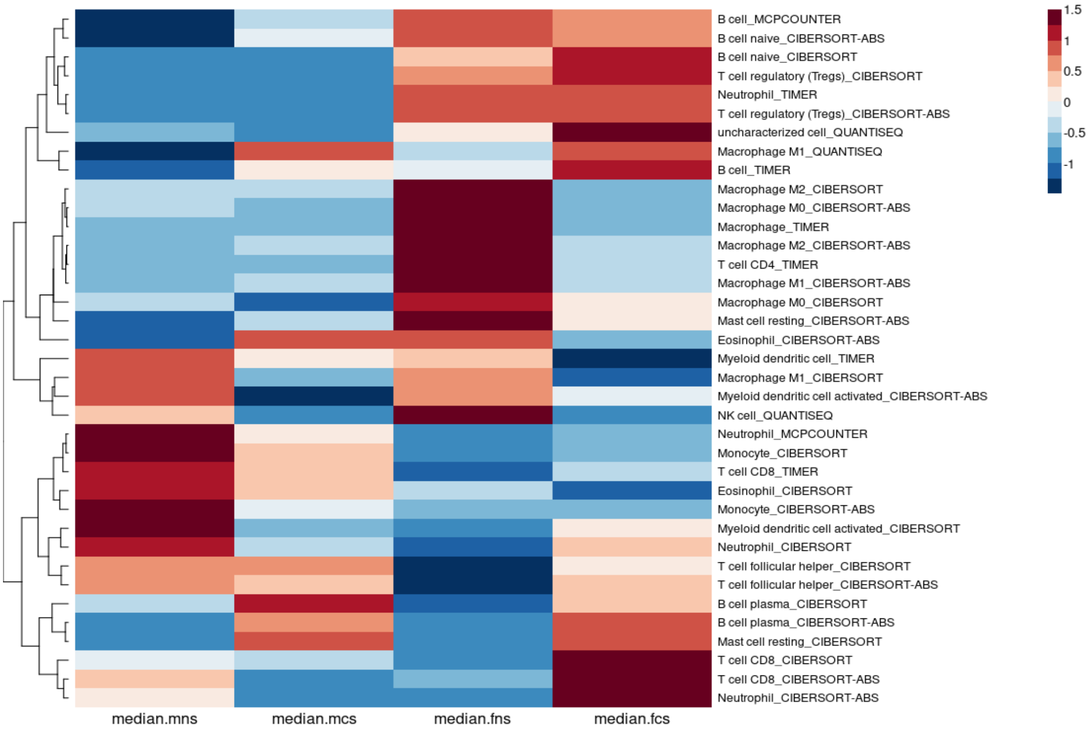

Supplementary Figure S4. Correlation of DEGs mRNA expression with immune cell infiltrates from TIMER 2.0 in LUAD patient groups according to smoking status and sex.

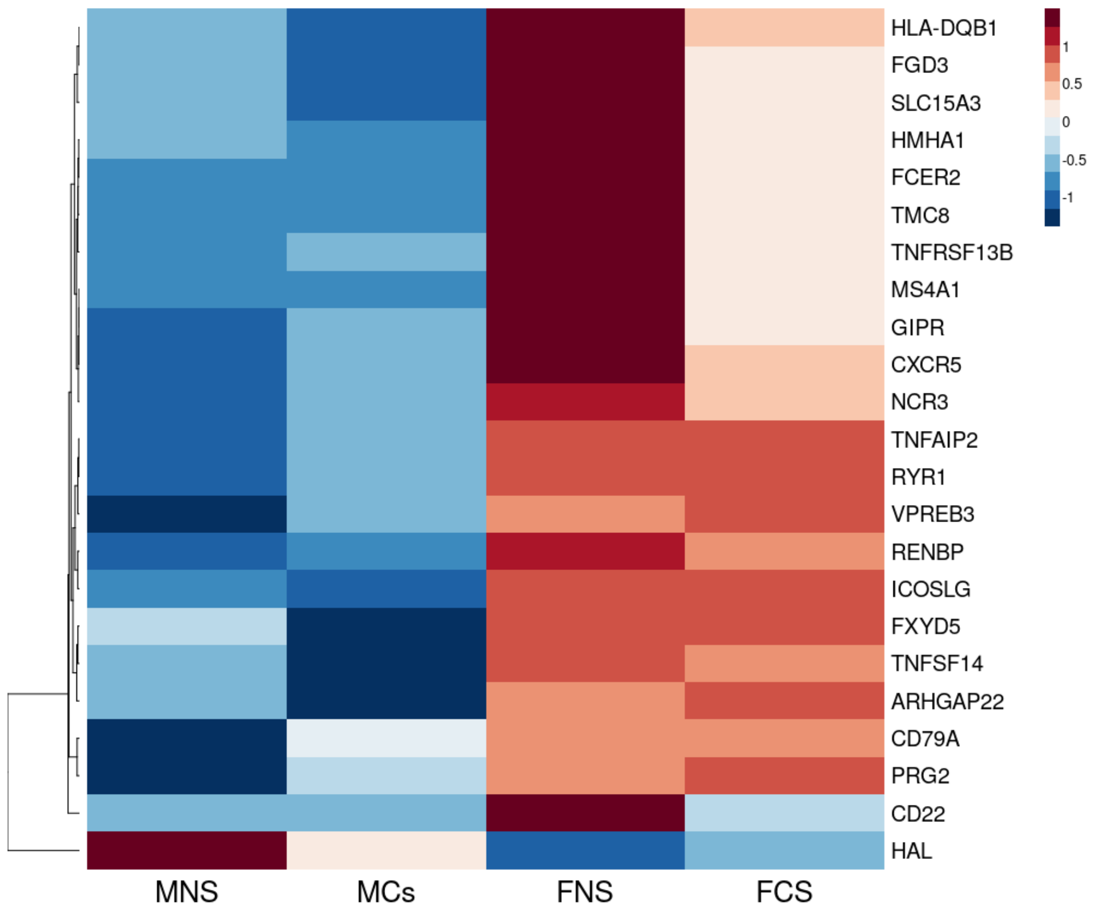

Supplementary Figure S5. Expression of immune related DEGs in LUAD patient groups according to smoking status and sex.

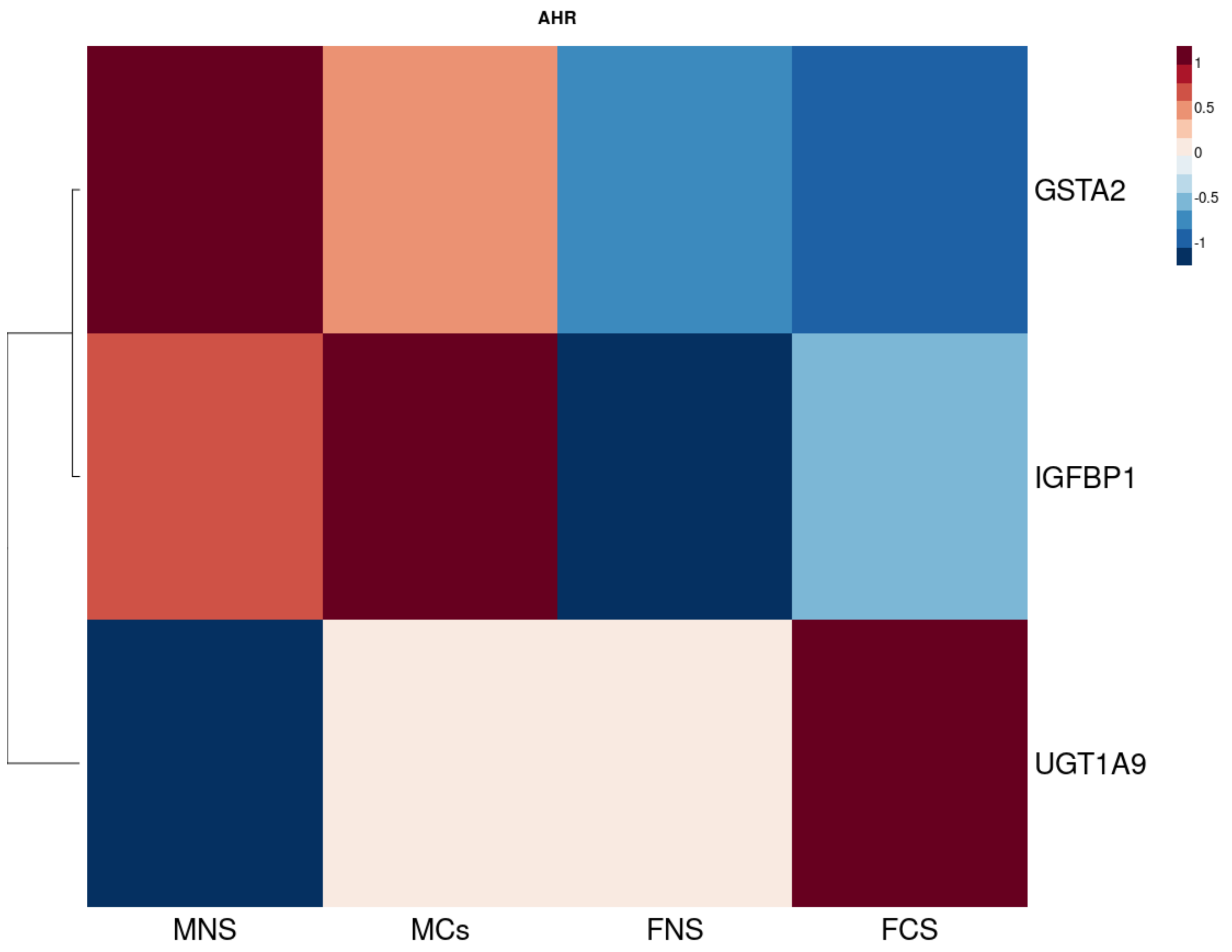

Supplementary Figure S6. Expression of detoxification related genes s in LUAD patient groups according to smoking status and sex.
